# Identifying critical risk factors in railway operations based on directed weighted complex networks and combinatorial weighting-TOPSIS

**DOI:** 10.1371/journal.pone.0357901

**Published:** 2026-09-10

**Authors:** Guanyi Liu, Xuewei Li, Rui Yang

**Affiliations:** 1 Transportation and Economic Research Institute, China Academy of Railway Sciences Corporation Limited, Beijing, China; 2 School of Economics and Management, Beijing Jiaotong University, Beijing, China; Southwest Jiaotong University, CHINA

## Abstract

The increasingly complex nature of modern railway operations has led to frequent accidents. Identifying the root causes of these accidents is essential for effective risk control. However, traditional risk identification methods are no longer adequate for handling such complexity. This paper introduces a novel approach for identifying key risk factors and performing a dynamic analysis of railway operation accidents based on a directed weighted complex network. First, risk factors and causal relationships were extracted from 168 railway accident reports based on accident chain theory to construct a Directed Weighted Railway Operation Accidents Complex Network (ROACN). Second, to identify key nodes within the network, a comprehensive node importance evaluation method based on combination weighting-TOPSIS was developed. Finally, a dynamic simulation analysis of network robustness was conducted under seven different targeted attack strategies. The results demonstrate that the ROACN exhibits scale-free network characteristics. Furthermore, attacking the ROACN based on the proposed comprehensive node importance index revealed that the network possesses excellent robustness, indicating that the proposed method reflects key node importance more accurately than traditional evaluation indicators. Consequently, implementing targeted interventions for these key risk nodes can effectively mitigate the propagation of risks and reduce the occurrence of railway accidents.

## Introduction

Railway operations play an essential role in economic and social development. With the continuous growth in transportation demand and the increasing density of railway networks, significant challenges have been posed to operational safety. Accidents involving high-speed trains, in particular, often result in substantial economic losses and severe casualties. Consequently, railway operational safety has garnered significant national and international attention [1]. Many countries have progressively integrated emerging technologies—such as big data, cloud computing, the Internet of Things (IoT), and 5G—into railway management to enhance the efficiency of transportation systems. However, the operational risks inherent in train operations are characterized by latency, coupling, and cascading effects. Despite notable advancements in risk prevention and safety management, the frequency of railway accidents remains unsatisfactory compared to expected safety levels [2]. Railway accidents often stem from a myriad of factors, including human error, equipment failure, harsh environmental conditions, and inadequate management. These factors frequently interact, compounding the overall likelihood of an accident. Analyses of official accident reports indicate that most railway incidents are attributable to the coupling of multiple risk factors. As illustrated by the domino effect, a minor shift in the initial state of one event within a interconnected series can trigger a cascading failure [3]. During railway transportation, a state change in a specific factor can propagate risk to adjacent nodes via connecting edges, ultimately culminating in an accident and forming a complete accident chain. Distinct accidents generate disparate accident chains, which collectively constitute complex accident networks. Crucially, accurately identifying key factors in railway accidents and analyzing their risk evolution paths is vital for ensuring the safety of railway operations.

Given that railway operational accidents typically result in catastrophic economic losses and casualties, accident prevention has become a critical research paradigm. Risk-based analysis, in particular, has received extensive attention, leading to various theoretical models and risk assessment methods. However, traditional risk analysis methods primarily utilize linear causality to describe static dependencies between logical variables. These methods are typically restricted to single systems or isolated analysis objects, which limits their effectiveness when applied to complex, interconnected railway operation systems [4]. Additionally, while Bayesian networks have been employed to analyze accident risks, they rely heavily on expert estimations for accident probabilities, thus lacking a mechanism to identify high-risk events based on underlying accident physics. Recently, complex network theory has emerged as a mainstream methodology for accident analysis. By characterizing risk factors and their interrelationships, complex network models enable the effective identification of critical risks. Nevertheless, current research on railway operational accidents utilizing complex network theory still exhibits three critical deficiencies:

(1) Few studies have analyzed railway accident networks from the explicit perspective of the accident chain, leaving the structural importance of nodes in complex railway systems insufficiently discussed. Moreover, existing research primarily focuses on theoretical comparisons between basic network models, with limited consideration given to solving actual engineering problems.
(2) Previous studies evaluating node importance have predominantly relied on single indicators without adequately considering the overall network structure and performance. Notably, existing node evaluation metrics rarely account for nodes that occupy critical “structural hole” positions.
(3) Current literature focuses heavily on static network robustness following the failure of a single node, largely overlooking dynamic network robustness under continuous, sequential node failures.

In response to these limitations, this paper accounts for the complexity, coupling, and cascading characteristics of railway operational accidents by integrating accident chain theory with complex network theory. A novel node importance evaluation method is proposed, and the dynamic robustness of the network is systematically analyzed. The main contributions of this study are summarized as follows:

(1) Diverging from traditional undirected or unweighted risk networks, this study constructs a directed weighted railway operation accident complex network (ROACN). Grounded in real-world railway accident reports, this network identifies risk factors and their interrelationships through accident chain theory, thereby preventing structural topology failures.
(2) To overcome the subjective bias or lack of professional domain expertise inherent in traditional node importance assessment frameworks, this study adopts a combination optimization calculation method based on game theory. Furthermore, the incorporation of structural hole indicators enables a more comprehensive evaluation of network topological characteristics.
(3) This study develops a dynamic node fault algorithm that advances beyond static robustness evaluations. By continuously updating the node importance rankings, this method captures the dynamic evolution of the network structure under attack. The simulation results successfully demonstrate the effectiveness and accuracy of the proposed node importance calculation method.

The remainder of this paper is structured as follows: Section 2 briefly reviews related work. Section 3 presents the proposed methodology. Section 4 details the construction of the ROACN and analyzes node importance under different evaluation metrics. Section 5 calculates and discusses the network structural indicators and node importance. Section 6 investigates the dynamic robustness of the network under various attack strategies, verifying the effectiveness of the proposed method. Finally, Section 7 presents the conclusions of this study

## Related works

### Accident risk analysis based on complex networks

Accident causation analysis plays a fundamental role in understanding the mechanisms underlying accident occurrence and propagation. Existing accident analysis approaches can generally be classified into four categories: system-based models, sequential models, epidemic-spreading models, and network-based models. System-based methods, such as the Functional Resonance Analysis Method (FRAM) [5,6], System-Theoretic Accident Model and Processes (STAMP) [7,8], and Human Factors Analysis and Classification System (HFACS) [9,10], emphasize interactions among organizational, technical, and human factors. Sequential models, including Fault Tree Analysis (FTA) [11,12] and Event Tree Analysis (ETA) [13,14], focus on linear causal chains of accident evolution. Epidemic-spreading models, such as the SIR, describe the diffusion characteristics of risk within complex systems [15,16]. However, these methods often struggle to capture the nonlinear interactions, cascading effects, and coupling relationships among multiple accident factors.

To overcome these limitations, complex network theory has increasingly been adopted for accident causation analysis due to its ability to characterize heterogeneous interactions among risk factors and reveal the structural properties of accident systems. Existing studies have applied complex networks to various engineering domains, including maritime transportation, hazardous materials transportation, coal mining, urban rail transit, and construction safety. For example, Deng et al. [17] analyzed the structural characteristics of maritime traffic accident networks, while Noguchi et al. [18] explored multi-scenario risk propagation in hazardous materials transportation through network-based accident modeling. Zhang et al [19] proposed a complex network-based causal model of human error for human-caused risk analysis of gas explosion accidents in China. In the field of transportation safety, Zhou et al. [20] established a directed accident causation network for railway accidents and identified critical accident chains through topological analysis. Similarly, Wang et al. [21] developed a causation network for urban rail transit accidents, and Miao et al. constructed a coal mine roof accident network to identify critical risk factors using robustness analysis.

Although these studies demonstrate the effectiveness of complex networks in uncovering accident causation mechanisms, their methodological focuses differ considerably. Some studies primarily investigate network topology and structural characteristics, whereas others emphasize risk propagation paths or critical factor identification [22–25]. Moreover, most existing accident networks are constructed as static representations of accident systems and mainly rely on unweighted or partially weighted relationships among factors. Such simplifications may lead to information loss regarding causal strength and accident evolution processes.

More importantly, railway operation accidents exhibit strong sequential dependence and dynamic propagation characteristics. Accident factors are often connected through accident chains, where causal influences possess both directionality and varying interaction strengths. Existing studies seldom integrate these characteristics into a unified directed and weighted complex network framework. Consequently, the dynamic transmission mechanisms of accident risks and the relative importance of accident factors may not be adequately captured.

### Node importance evaluation methods

Identifying critical nodes is a central issue in complex network analysis because network performance and risk propagation are often highly dependent on a limited number of influential nodes. Existing node importance evaluation methods can be broadly categorized into topology-based measures, multi-indicator evaluation approaches, and risk-oriented assessment methods [26].

Topology-based indicators, including degree centrality, betweenness centrality, closeness centrality, and eigenvector centrality, are the most widely used approaches. Degree centrality identifies highly connected nodes, whereas betweenness centrality highlights nodes acting as bridges in information transmission. Closeness centrality evaluates the accessibility of nodes within the network, while eigenvector centrality considers both direct and indirect influences. These indicators have been extensively applied in transportation, infrastructure, and safety networks to identify critical components and vulnerable locations [27].

However, relying on a single topological indicator often produces biased results because different metrics capture different aspects of node influence [28]. To overcome this limitation, several researchers have proposed multi-criteria evaluation frameworks. For instance, Faramondi et al. [29] integrated multiple centrality measures into a comprehensive assessment framework for infrastructure networks. Zhang and Geng [30] proposed a comprehensive index system for identifying key factors in electrical accident networks. Feng et al. [31] developed a dynamic node importance evaluation model considering network evolution characteristics.

In parallel, risk-oriented approaches have emerged for accident analysis applications. These methods incorporate accident causation mechanisms [32], risk propagation characteristics [33], and domain-specific knowledge into node importance assessment [34]. Liu et al. [35] utilized knowledge graphs to identify critical risk factors in railway accident systems, whereas and Li et al. [36] employed Bayesian-network-based approaches to evaluate accident causation importance under uncertainty. Compared with purely topological methods, these approaches provide richer semantic interpretations but often require extensive prior knowledge and complex parameter estimation [37].

Despite these advances, several limitations remain. First, many existing studies evaluate node importance using only one or two indicators, potentially overlooking the multidimensional nature of node influence. Second, most methods focus on conventional centrality measures while neglecting structural hole characteristics, which are crucial for identifying nodes that control information flow between network communities. Third, evaluation frameworks developed for general-purpose networks may not adequately capture the structural characteristics of railway accident networks.

### Complex network robustness analysis

Network robustness refers to the ability of a network to maintain its structural integrity and functional performance when subjected to failures or external attacks [38]. Robustness analysis has become an important research topic because it provides insights into network vulnerability, resilience, and risk mitigation strategies [39].

Existing robustness studies mainly focus on transportation, infrastructure, social, and energy networks [40]. Transportation-related research has investigated the robustness of air transport networks, multimodal freight systems, and urban transportation systems under different disruption scenarios. For instance, Mattsson and Jenelius [41] proposed the concept of resilience to provide a framework for the ability of transport systems to maintain or quickly recover its functions. These studies generally evaluate network performance degradation under random failures or targeted attacks and employ indicators such as network efficiency, connectivity, and giant component size. Infrastructure-related studies have further extended robustness analysis to weighted and coupled networks, considering cascading failures and interdependent relationships among network components [42–45].

From a methodological perspective, current robustness analysis approaches can be divided into static and dynamic frameworks. Static robustness assessment typically evaluates network responses to individual node or edge removals. Dynamic robustness analysis, in contrast, considers continuous failures and cascading processes, thereby providing a more realistic representation of risk propagation in complex systems. For example, Wang et al. [46] took the inherent features of power grids into account and developed a novel multi-perspective framework for evaluating power network robustness. Chen et al. [47] established a network model for China’s crude oil import system. They validated the network’s robustness facing node malfunction via random and targeted attack simulations. Recent studies have demonstrated that dynamic failure mechanisms can significantly alter network vulnerability patterns compared with static analyses [48,49].

Nevertheless, several research gaps remain. First, most robustness studies focus on generic transportation or infrastructure networks rather than accident causation networks. Second, existing railway-related studies rarely consider directed weighted accident networks, despite the fact that accident causation relationships inherently possess directional and weighted characteristics. Third, robustness evaluations are predominantly conducted under single-failure scenarios, while continuous node failures and dynamic degradation processes are insufficiently explored.

## Methodology

**Fig 1:**
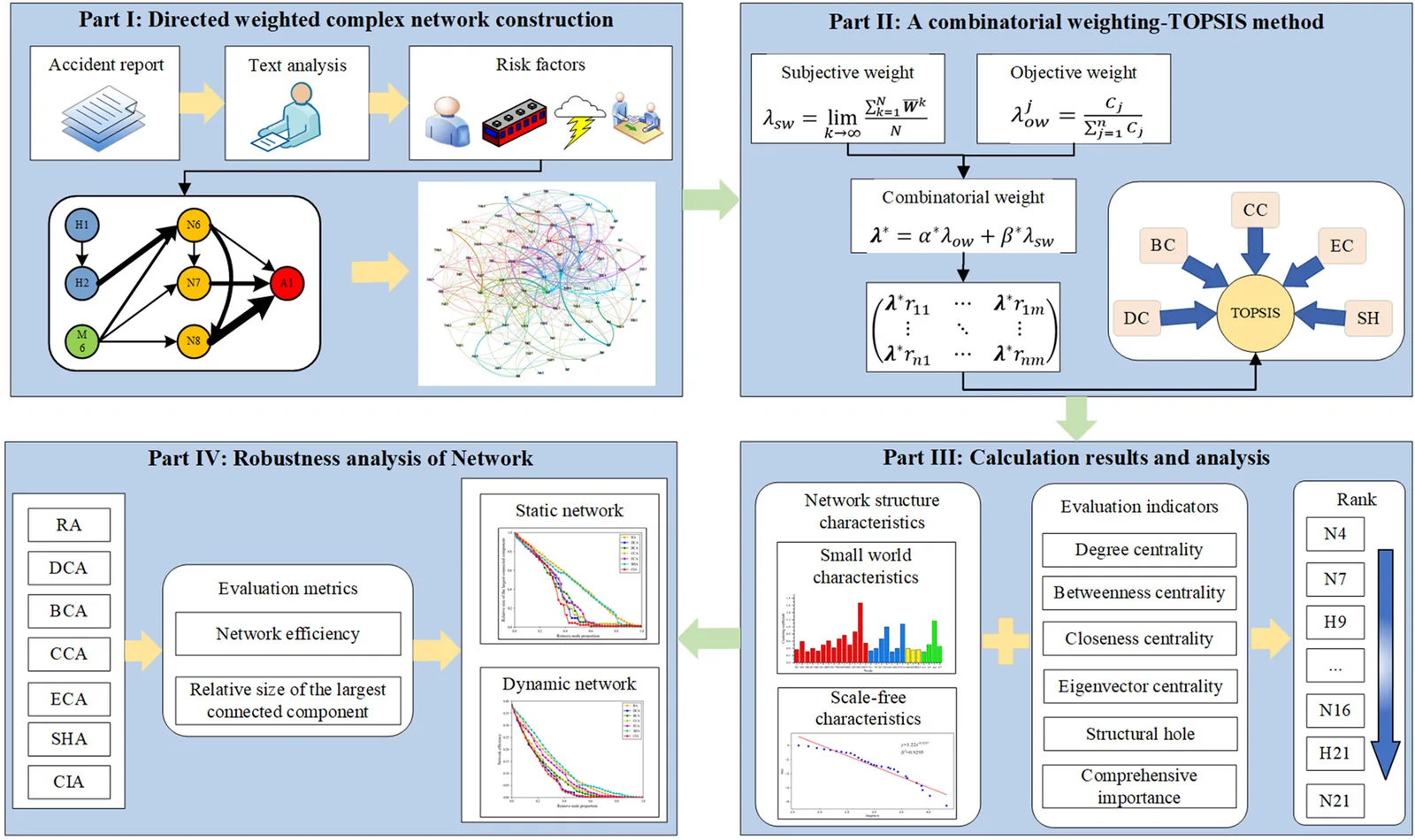
The framework of the proposed methodology.

Fig 1 shows the framework of the proposed methodology. There are four parts: Part I is directed weighted complex network construction. Part II is combinatorial weighting-TOPSIS method, Part III is topology analysis and calculation results. Part IV is robustness analysis of network. Additionally, we have invited five experts in the field of railway accident risk, including two professors from Beijing Jiaotong University, with a research focus on railway operation safety; two senior engineers from National Railway Group Co., Ltd. with rich experience in railway operations; one is a high-speed railway dispatch director with over 20 years of emergency management experience. The main task of the 5 experts is to handle the subjective weight of each indicator. We communicated via email and obtained their written consent.

### Directed weighted complex network

Complex networks (CN) can effectively analyze the coupling relationships and propagation pathways between different risk factors. Assuming a directed weighted complex network is expressed as G=(V,E), where $V=\left\{v_{\text{1}},v_{\text{2}},\ldots ,v_{n}\right\}$ represents the set of all nodes in the network, and $E=\left\{e_{\text{1}},e_{\text{2}},\ldots ,e_{n}\right\}$ represents the set of all edges in the network. A complex network can be described using an adjacency matrix, which is formulated as follows:


$$\begin{array}{c} \begin{array}{c} A_{ij}=\left\{ \begin{array}{c} @c\omega_{ij}\times a_{ij}\\ \text{0} \end{array}\right. \end{array} \end{array}$$ (1)

where $a_{ij}$ represents the directed edge from node i to node j. When $a_{ij}=\text{1}$ indicates the edge exists; otherwise $a_{ij}=\text{0}$ means the edge is absent. $\omega_{ij}$ denotes the weight of the edge between node i and node j, which represents the frequency of risk factor i triggering risk factor j. The risk factors involved in railway operation accidents have varying degrees of impact on the outcomes of these accidents. To better explore the risks associated with railway operation accidents, a directed weighted network is developed using the accident chain theory.

### Characteristic parameters of complex network

Each network exhibits specific topological features that describe its connectivity, interactions and dynamic processes. Analyzing the topological characteristics can effectively identify the key nodes in the network and their dynamic interactions. The key indicators used to evaluate the topological characteristics of the network are as follows.

#### Node degree.

Node degree reflects the importance of the node in the network. The greater the order of a node, the more nodes it affects, indicating that the node is more important. The number of edges connecting node i to other nodes is defined as the order of node i and is degraded by D(i). In directed weighted networks, the degree of a node is divided into out-degree and in-degree. which can be defined as:


$$\begin{array}{c} D(i)=\sum_{j\in V}a_{ij}+\sum_{j\in V}a_{ji} \end{array}$$ (2)

$$\begin{array}{c} D_{in}(i)=\sum_{j\in V}a_{ji} \end{array}$$ (3)

$$\begin{array}{c} D_{out}(i)=\sum_{j\in V}a_{ij} \end{array}$$ (4)

where $D_{out}$ denotes the number of edges from node i to other nodes. $D_{in}$ denotes the number of edges from other nodes to node i.

#### Average path length.

Average path length is the average number of steps along the shortest path from any node to another node. Thus, it quantifies the degree of separation between the nodes of the network. The average path length L of a network can be calculated as:


$$\begin{array}{c} L=\frac{\text{1}}{N(N-\text{1})}\sum_{i\neq j}l_{ij} \end{array}$$ (5)

where N denotes the number of nodes in the network. $l_{ij}$ denotes the number of edges on the shortest path between any two nodes i and j in the network.

#### Clustering coefficient.

Clustering coefficient is an attribute that describes the degree of aggregation of nodes within the network. The clustering coefficient $C_{i}$ of node i is calculated as follows:


$$\begin{array}{c} \begin{array}{c} C_{i}=\frac{\text{2}E_{i}}{k_{i}\left(k_{i}-\text{1}\right)} \end{array} \end{array}$$ (6)

where $E_{i}$ denotes the number of connected edges between node i and all its neighbouring nodes. If the clustering coefficient of the node takes a higher value, it means that the surrounding nodes are more connected to the node.

The clustering coefficient C of the whole network is the average of the clustering coefficients of all the nodes with the following expression:


$$\begin{array}{c} \begin{array}{c} C=\frac{\sum_{i=\text{1}}^{N}C_{i}}{N} \end{array} \end{array}$$ (7)

#### Betweenness centrality.

The betweenness of a node refers to the number of shortest paths that pass through the node in the network, indicating the importance of the node in transmitting information in the network structure. The larger the betweenness of a node, the stronger its ability to transfer information, and the closer the node is to the center in the network. The betweenness centrality of node i is calculated as follows:


$$\begin{array}{c} B_{i}=\sum_{i\neq s\neq j}\frac{Q_{s}(i,j)}{Q(i,j)} \end{array}$$ (8)

where $Q_{s}(i,j)$ represents the number of shortest paths passing through node s. Q(i,j) represents the number of shortest paths between node i and node j.

#### Closeness centrality.

Closeness centrality indicates the extent to which a node is situated at the center of a network. If a node is interconnected with all other nodes and the distances between them are short, it suggests that the node is at the center of the network. The closeness centrality of node i is calculated as follows:


$$\begin{array}{c} \begin{array}{c} {CC}_{in}(i)=\frac{n-\text{1}}{\sum_{j=\text{1}}^{n}d_{ji}} \end{array} \end{array}$$ (9)

$$\begin{array}{c} \begin{array}{c} {CC}_{out}(i)=\frac{n-\text{1}}{\sum_{j=\text{1}}^{n}d_{ij}} \end{array} \end{array}$$ (10)

where ${CC}_{in}(i)$ denotes the in-closeness centrality of node i, ${CC}_{out}(i)$ denotes the out- closeness centrality of node i. $d_{ij}$ represents the shortest path distance from node i to node j. n−1 represents the maximum possible number of adjacent nodes in the network.

#### Structural hole.

Structural hole is a concept introduced by Burt in the study of competitive relations in social networks. The theory suggests that if there is no direct connection between two nodes in a network and a connection can only be established through a third node, then the third node occupies a structural hole in the network. This node has an information and control advantage over the other two nodes. Burt proposed measuring structural holes by calculating the network constraint coefficient. Constraint coefficient of structural hole of node i is calculated for is as follows:


$$\begin{array}{c} C_{i}=\sum_{j}\;{\left(P_{ij}+\sum_{q\neq i\neq j}\;P_{iq}P_{qj}\right)}^{\text{2}} \end{array}$$ (11)

$$\begin{array}{c} \begin{array}{c} p_{ij}=\frac{z_{ij}}{\sum_{j\in \Gamma (i)}\;z_{ij}} \end{array} \end{array}$$ (12)

$$\begin{array}{c} \begin{array}{c} z_{ij}=\left\{ \begin{array}{c} \text{1}\text{ Nodes }i,j\text{ are connected}\\ \text{0}\text{ Nodes }i,j\text{ are not connected} \end{array}\right. \end{array} \end{array}$$ (13)

where q represents an indirect node that connects node i to node j. $P_{ij}$ represents the proportion of energy spent by node i on node j to its total energy.

#### Eigenvector centrality.

Eigenvector centrality is one of the key indicators for evaluating the importance of nodes in complex networks. The principle behind eigenvector centrality is that the importance of a node is not only determined by the number of its connections, but also by the importance of each of those connections. A node with connections to other high-importance nodes is itself more important. Let $x_{i}$ represent the importance of node i, then the eigenvector centrality EC(i) is calculated as follows:


$$\begin{array}{c} EC(i)=x_{i}=\frac{\text{1}}{\lambda }\sum_{j=\text{1}}^{n}a_{ij}X_{j} \end{array}$$ (14)

where λ represents the eigenvalue of the adjacency matrixA. $a_{ij}$ denotes the node loss from node i to node j. $A=\left(a_{ij}\right)$ denotes the adjacency matrix of the network. $X={\left[X_{\text{1}}X_{\text{2}}\ldots X_{n}\right]}^{T}$ represents the eigenvector corresponding to the largest eigenvalue of the adjacency matrixA.

### Combinatorial weighting-TOPSIS method

#### TOPSIS method.

Based on the Technique for Order of Preference by Similarity to Ideal Solution (TOPSIS) method, the comprehensive evaluation of node importance is conceptualized by treating each node in a complex network as an alternative scheme, while the various indicators used to assess node importance are regarded as the attributes of these schemes. This formulation transforms the evaluation of node importance into a multi-attribute decision-making problem, where the decision criterion is the degree of importance of each scheme within the complex network [50].

Assuming that there are n nodes in the complex network, the corresponding set of alternative schemes can be expressed as $X=\left\{X_{\text{1}},X_{\text{2}},\ldots ,X_{n}\right\}$. If there are m indicators for evaluating the importance of nodes, the numerical combination of the corresponding schemes can be expressed as $Y=\left\{Y_{\text{1}},Y_{\text{2}},\ldots ,Y_{m}\right\}$. Therefore, the value of the j−th indicator of the i−th node is written as $X_{i}\left(Y_{j}\right)$, where i=1,2,…,n;j=1,2,…,m.

Step 1: The initial decision matrix A is formulated as follows:


$$\begin{array}{c} \begin{array}{c} A=(\begin{array}{ccc} X_{\text{1}}\left(Y_{\text{1}}\right) & \cdots & X_{\text{1}}\left(Y_{m}\right)\\ \vdots & \ddots & \vdots \\ X_{n}\left(Y_{\text{1}}\right) & \cdots & X_{n}\left(Y_{m}\right) \end{array}) \end{array} \end{array}$$ (15)

Step 2: Due to the multitude of evaluation indicators, potential interactions and scaling differences exist among them. Indicators are categorized into two types: benefit indicators and cost indicators. To eliminate the impact of different physical dimensions, these indicators must be standardized.

For a benefit indicator, the standardized value $r_{ij}$ is defined as:


$$\begin{array}{c} \begin{array}{c} r_{ij}=\frac{X_{i}\left(Y_{j}\right)}{X_{i}{\left(Y_{j}\right)}^{max}} \end{array} \end{array}$$ (16)

For a cost indicator, the standardized value $r_{ij}$ is defined as:


$$\begin{array}{c} \begin{array}{c} r_{ij}=\frac{X_{i}{\left(Y_{j}\right)}^{\text{min}}}{X_{i}\left(Y_{j}\right)} \end{array} \end{array}$$ (17)

Consequently, the normalized decision matrix R is obtained as:


$$\begin{array}{c} \begin{array}{c} R=(\begin{array}{ccc} r_{\text{1}\text{1}} & \cdots & r_{\text{1}m}\\ \vdots & \ddots & \vdots \\ r_{n\text{1}} & \cdots & r_{nm} \end{array}) \end{array} \end{array}$$ (18)

Step 3: Assume the weight of j−th indicator is $\omega_{j}$, where $j=\text{1},\text{2},\ldots ,m,\sum \omega_{j}=\text{1}$. The weights of the different indicators are brought to the normalized decision matrix R and a weighted normalized matrix $\overset{―}{R}$ is obtained, which can be calculated as:


$$\begin{array}{c} \begin{array}{c} \overset{―}{R}=\left(\overset{―}{r_{ij}}\right)=\left(w_{j}r_{ij}\right)=(\begin{array}{ccc} w_{\text{1}}r_{\text{1}\text{1}} & \cdots & w_{m}r_{\text{1}m}\\ \vdots & \ddots & \vdots \\ w_{\text{1}}r_{n\text{1}} & \cdots & w_{m}r_{nm} \end{array}) \end{array} \end{array}$$ (19)

Step 4: Determine the positive ideal decision-making scheme $X^{+}$ and the negative ideal decision-making scheme $X^{-}$ based on the matrix $\overset{―}{R}$, which can be calculated as:


$$\begin{array}{c} \begin{array}{c} X^{+}=\left\{\underset{i\in n}{max}\;\left(\overset{―}{r_{i\text{1}}},\cdots ,\overset{―}{r_{im}}\right)\right\}=\left\{{\overset{―}{r}}_{\text{1}}^{max},\cdots ,{\overset{―}{r}}_{m}^{max}\right\} \end{array} \end{array}$$ (20)

$$\begin{array}{c} \begin{array}{c} X^{-}=\left\{\underset{i\in n}{min}\;\left(\overset{―}{r_{i\text{1}}},\cdots ,\overset{―}{r_{im}}\right)\right\}=\left\{{\overset{―}{r}}_{\text{1}}^{min},\cdots ,{\overset{―}{r}}_{m}^{min}\right\} \end{array} \end{array}$$ (21)

Step 5: Calculate the distance from each scheme $X_{i}$ to the positive ideal scheme $X^{+}$ and the negative ideal scheme $X^{-}$, which can be calculated as:


$$\begin{array}{c} D_{i}^{+}=\sqrt{\sum_{j=\text{1}}^{m}\;{\left(\overset{―}{r_{ij}}-{\overset{―}{r}}_{j}^{max}\right)}^{\text{2}}} \end{array}$$ (22)

$$\begin{array}{c} D_{i}^{-}=\sqrt{\sum_{j=\text{1}}^{m}\;{\left(\overset{―}{r_{ij}}-y_{j}^{min}\right)}^{\text{2}}} \end{array}$$ (23)

Step 6: Calculate the proximity of the ideal scheme. Rank the importance according to the value of proximity $Z_{i}$, complete the node importance evaluation. The expression of $Z_{i}$ as follows:


$$\begin{array}{c} \begin{array}{c} Z_{i}=\frac{D_{i}^{-}}{\left(D_{i}^{-}+D_{i}^{+}\right)}({\text{0}\leq Z}_{i}\leq \text{1}) \end{array} \end{array}$$ (24)

where $Z_{i}$ indicates the importance of node i, and its value ranges from ${\text{0}\leq Z}_{i}\leq \text{1}$. A larger value of $Z_{i}$ indicates a higher importance of node i.

#### Subjective weight confirmation based on ANP.

The Analytic Hierarchy Process (AHP) has been extensively utilized to subjectively determine indicator weights in complex networks. Relying on expert domain knowledge and practical experience, AHP assigns weightings to individual indicators. However, it disregards their mutual correlations. To address this, the Analytic Network Process (ANP) is proposed by Saaty as an extension of AHP. As an advanced decision-making methodology, ANP retains the advantages of AHP while accounting for interdependencies and feedback loops among elements or clusters within the network. ANP utilizes a supermatrix to comprehensively analyze interacting factors, thereby deriving final hybrid weights. Given its robust decision-making capacity, ANP has been widely applied to complex system problems [51]. In this study, ANP is employed to calculate the subjective weights through the following steps.

Step 1: Construct the judgement matrix under the control layer criterion. After consistency check, the local weight vector matrix $W_{ij}$ is constructed. The calculation formula is as follows:


$$\begin{array}{c} \begin{array}{c} W_{ij}=[\begin{array}{ccc} \begin{array}{cc} \begin{array}{c} w_{\text{1}\text{1}}\\ w_{\text{2}\text{1}} \end{array} & \begin{array}{c} w_{\text{1}\text{2}}\\ w_{\text{2}\text{2}} \end{array} \end{array} & \begin{array}{c} \cdots \\ \cdots \end{array} & \begin{array}{c} w_{\text{1}n}\\ w_{\text{2}n} \end{array}\\ \vdots & \ddots & \vdots \\ \begin{array}{cc} w_{n\text{1}} & w_{n\text{2}} \end{array} & \cdots & w_{nn} \end{array}] \end{array} \end{array}$$ (25)

Step 2: Build a supermatrix to construct local vector matrix for all factors, normalize the eigenvectors corresponding to the largest eigenvalue, and combine all eigenvectors to form a supermatrix $W^{\prime }$, which can be calculated as:


$$\begin{array}{c} \begin{array}{c} W^{\prime }=[\begin{array}{ccc} \begin{array}{cc} \begin{array}{c} W_{\text{11}}\\ W_{\text{21}} \end{array} & \begin{array}{c} W_{\text{12}}\\ W_{\text{22}} \end{array} \end{array} & \begin{array}{c} \cdots \\ \cdots \end{array} & \begin{array}{c} W_{\text{1}N}\\ W_{\text{2}N} \end{array}\\ \vdots & \ddots & \vdots \\ \begin{array}{cc} W_{N\text{1}} & W_{N\text{2}} \end{array} & \cdots & W_{NN} \end{array}] \end{array} \end{array}$$ (26)

Step 3: Under the control layer criterion, each factor of the network layer is compared in terms of its importance to the different indicators to obtain the normalized ranking vector $L_{j}=\left[l_{\text{1}j},l_{\text{2}j},\ldots ,l_{Mj}\right]$, which in turn yields the weighted supermatrix L which is calculated as:


$$\begin{array}{c} \begin{array}{c} L=[\begin{array}{ccc} \begin{array}{cc} \begin{array}{c} l_{\text{11}}\\ l_{\text{21}} \end{array} & \begin{array}{c} l_{\text{12}}\\ l_{\text{22}} \end{array} \end{array} & \begin{array}{c} \cdots \\ \cdots \end{array} & \begin{array}{c} l_{\text{1}N}\\ l_{\text{2}N} \end{array}\\ \vdots & \ddots & \vdots \\ \begin{array}{cc} l_{M\text{1}} & l_{M\text{2}} \end{array} & \cdots & l_{MN} \end{array}] \end{array} \end{array}$$ (27)

The weighted hypermatrix is obtained by weighting the hypermatrix. Its expression is as follows:


$$\begin{array}{c} \begin{array}{c} \overset{―}{W}=LW^{\prime }=[\begin{array}{ccc} \begin{array}{cc} \begin{array}{c} l_{\text{11}}W_{\text{11}}\\ l_{\text{21}}W_{\text{21}} \end{array} & \begin{array}{c} l_{\text{12}}W_{\text{12}}\\ l_{\text{22}W_{\text{22}}} \end{array} \end{array} & \begin{array}{c} \cdots \\ \cdots \end{array} & \begin{array}{c} l_{\text{1}N}W_{\text{1}N}\\ l_{\text{2}N}W_{\text{2}N} \end{array}\\ \vdots & \ddots & \vdots \\ \begin{array}{cc} l_{N\text{1}}W_{N\text{1}} & l_{N\text{2}} \end{array}W_{N\text{2}} & \cdots & l_{NN}W_{NN} \end{array}] \end{array} \end{array}$$ (28)

Step 4: The local weight of each indicator under the guideline is determined by calculating the limit value of the weighted supermatrix $\overset{―}{W}$, the subjective weight $\lambda_{sw}$ of each indicator to the overall goal is calculated as follows:


$$\begin{array}{c} \begin{array}{c} W^{\infty }=\underset{k\to \infty }{\text{lim}}\frac{\sum_{k=\text{1}}^{N}{\overset{―}{W}}^{k}}{N} \end{array} \end{array}$$ (29)

#### Objective weight confirmation based on CRITIC.

The CRITIC method, proposed by Diakoulaki in 1995, is an objective weighting approach driven purely by indicator data [52]. Compared to the entropy weight method or standard deviation method, CRITIC delivers a superior objective weighting scheme by simultaneously accounting for the information intensity within indicators and the conflict intensity among them. Contrast intensity, expressed via the standard deviation, reflects the degree of value variation across different alternatives for a given indicator; a higher standard deviation denotes larger fluctuations, thereby warranting a higher weight. Conflict intensity is captured via correlation coefficients; a strong positive correlation between two indicators indicates lower conflict, thereby reducing the assigned weight. The execution steps are as follows.

Step 1: The processing method is divided into positive processing and negative processing. Assuming a total of m decision-making options, each option has n indicators, construct the initial indicator matrix X is shown as:


$$\begin{array}{c} \begin{array}{c} X=[\begin{array}{ccc} x_{\text{1}\text{1}} & \cdots & x_{\text{1}n}\\ \vdots & \ddots & \vdots \\ x_{m\text{1}} & \cdots & x_{mn} \end{array}] \end{array} \end{array}$$ (30)

The normalized indicators are denoted as $x_{ij}^{\prime }$. For benefit indicators, the standardization is defined as:


$$\begin{array}{c} \begin{array}{c} x_{ij}^{\prime }=\frac{x_{j}-x_{min}}{x_{max}-x_{min}} \end{array} \end{array}$$ (31)

For cost (or negative) indicators, the standardization is defined as:


$$\begin{array}{c} \begin{array}{c} x_{ij}^{\prime }=\frac{x_{max}-x_{j}}{x_{max}-x_{min}} \end{array} \end{array}$$ (32)

Step 2: Contrast refers to the magnitude of the difference between the values of the same indicator across evaluation schemes. The contrast of indicator j is denoted as $\sigma_{j}$, which is calculated as:


$$\begin{array}{c} \begin{array}{c} \sigma_{j}=\sqrt{\frac{\sum_{i=\text{1}}^{m}\left(x_{ij}^{\prime }-\overset{―}{x_{j}^{\prime }}\right)}{m-\text{1}}} \end{array} \end{array}$$ (33)

$$\begin{array}{c} \begin{array}{c} \overset{―}{x_{j}^{\prime }}=\frac{\text{1}}{m}\sum_{i=\text{1}}^{m}x_{ij}^{\prime } \end{array} \end{array}$$ (34)

Step 3: Contradiction is the degree of correlation between different indicators. If a significant positive correlation is presented, the smaller the value of contradiction is. Assuming that the contradiction between indicator j and the rest of the indicators is $f_{j}$, which is calculated as:


$$\begin{array}{c} \begin{array}{c} f_{j}=\sum_{i=\text{1}}^{m}\left(\text{1}-r_{ij}\right) \end{array} \end{array}$$ (35)

Step 4: Assume that the information carrying capacity of indicator j is $C_{j}$, which is calculated as:


$$\begin{array}{c} \begin{array}{c} C_{j}=\sigma_{j}\times f_{j} \end{array} \end{array}$$ (36)

Step 5: Based on the information carrying capacity of indicator j, the objective weight $\lambda_{ow}$ of the jth indicator can be obtained, which is calculated as:


$$\begin{array}{c} \begin{array}{c} \lambda_{ow}^{j}=\frac{C_{j}}{\sum_{j=\text{1}}^{n}C_{j}} \end{array} \end{array}$$ (37)

### Combinatorial weight confirmation based on game theory

Subjective weighting methods often introduce individual biases, whereas objective weighting methods fully exploit data distributions but fail to reflect decision-makers’ professional insights. To bridge this gap, this study integrates the ANP and CRITIC methods using game theory to reconcile conflicts and achieve mathematical consensus. This strategy enhances both the scientific rigor and empirical rationality of the resulting weights. The operational steps are as follow.:

Step 1: According to the subjective weights $\lambda_{sw}$ and objective weights $\lambda_{ow}$, construct a vector of indicator combination weights λ, which is calculated as:


$$\begin{array}{c} \begin{array}{c} \lambda =\alpha \lambda_{ow}+\beta \lambda_{sw}\\ =[\begin{array}{cc} \lambda_{ow}^{\text{1}} & \lambda_{sw}^{\text{1}}\\ \begin{array}{c} \lambda_{ow}^{\text{2}}\\ \begin{array}{c} \vdots \\ \lambda_{ow}^{n} \end{array} \end{array} & \begin{array}{c} \begin{array}{c} \lambda_{sw}^{\text{2}}\\ \vdots \end{array}\\ \lambda_{sw}^{n} \end{array} \end{array}][\begin{array}{c} \alpha \\ \beta \end{array}]=[\begin{array}{c} {\alpha \lambda }_{ow}^{\text{1}}+{\beta \lambda }_{sw}^{\text{1}}\\ \begin{array}{c} {\alpha \lambda }_{ow}^{\text{2}}+{\beta \lambda }_{sw}^{\text{2}}\\ \begin{array}{c} \vdots \\ {\alpha \lambda }_{ow}^{n}+{\beta \lambda }_{sw}^{n} \end{array} \end{array} \end{array}] \end{array} \end{array}$$ (38)

where n is the number of evaluation indicators. α and β are the coefficients of the linear combination of subjective weights $\lambda_{sw}$ and objective weights $\lambda_{ow}$, respectively.

Step 2: According to the idea of game theory, the optimal linear combination coefficients are sought with the objective of minimizing the sum of the differences between the indicator combination weights λ and the subjective weights $\lambda_{sw}$ and objective weights $\lambda_{ow}$ respectively. The constructed objective function is shown as:


$$\begin{array}{c} min\left({\left\|\lambda -\lambda_{ow}\right\|}_{\text{2}}+{\left\|\lambda -\lambda_{sw}\right\|}_{\text{2}}\right)=min\left({\left\|\alpha \lambda_{ow}+\beta \lambda_{sw}-\lambda_{ow}\right\|}_{\text{2}}+{\left\|\alpha \lambda_{ow}+\beta \lambda_{sw}-\lambda_{sw}\right\|}_{\text{2}}\right) \end{array}$$ (39)

$$\begin{array}{c} \begin{array}{c} @cs.t.\left\{ \begin{array}{c} @c\alpha +\beta =\text{1}\\ \alpha \geq \text{0},\beta \geq \text{0} \end{array}\right. \end{array} \end{array}$$ (40)

Step 3: According to the principle of matrix differentiation, the above equation is subjected to first-order derivation to satisfy the derivation conditions as follows:


$$\begin{array}{c} \begin{array}{c} @l\left\{ \begin{array}{c} @l\alpha \lambda_{ow}\lambda_{ow}^{\text{T}}+\beta \lambda_{ow}\lambda_{sw}^{\text{T}}=\lambda_{ow}\lambda_{ow}^{\text{T}}\\ \alpha \lambda_{sw}\lambda_{ow}^{\text{T}}+\beta \lambda_{sw}\lambda_{sw}^{\text{T}}=\lambda_{sw}\lambda_{sw}^{\text{T}} \end{array}\right. \end{array} \end{array}$$ (41)

Step 4: The resulting weight coefficients α and β are normalised to obtain the optimal combination of weight coefficients $\alpha^{*}$ and $\beta^{*}$, which are calculated as:


$$\begin{array}{c} \begin{array}{c} @l\left\{ \begin{array}{c} @l\alpha^{*}=\frac{\left|\alpha \right|}{\left|\alpha |+|\beta \right|}\\ \beta^{*}=\frac{\left|\beta \right|}{\left|\alpha |+|\beta \right|} \end{array}\right. \end{array} \end{array}$$ (42)

Step 5: Obtain the optimal combination weight $\lambda^{*}$, which is calculated as:


$$\begin{array}{c} \begin{array}{c} \lambda^{*}=\alpha^{*}\lambda_{ow}+\beta^{*}\lambda_{sw} \end{array} \end{array}$$ (43)

## Case study

### Data collection

In this study, official UK railway operational accident reports spanning from 2016 to 2022 were selected as the primary data source. The relevant accident dossiers were retrieved from the official website of the UK Rail Accident Investigation Branch (RAIB) (https://www.gov.uk/government/organisations/rail-accident-investigation-branch). Notably, the historical accident data recorded by the RAIB originally included metro and tram incidents, which were systematically excluded during the data cleaning phase. Rather than introducing selection bias, this filtering process aimed to ensure the methodological homogeneity of the research subjects. Substantial operational discrepancies exist between heavy railway systems and urban rail transit systems regarding operating speeds, interlocking controls, dispatching commands, and safety management paradigms. Modeling both systems within a single network framework would allow the specific high-risk profiles unique to rapid urban transit to distort the identification of mainline railway operational risks. Consequently, focusing exclusively on railway operational accidents yields a more realistic and accurate risk identification profile.

Through this selection process, a total of 168 distinct railway operational accident reports were compiled. Detailed content analysis of these reports allowed the accidents to be classified into 16 distinct categories, including derailment, collision, train sliding, and near-misses, as detailed in Table 1. Crucially, near-miss incidents were retained in the dataset, as they contain potentially critical causal factors that are invaluable for understanding the underlying mechanisms of railway accidents.

**Table 1:** Type of railway accident.

No.	Node	No.	Node	No.	Node
A1	Derailment	A2	Collision	A3	Train sliding
A4	Near miss	A5	Overspeed	A6	Drag
A7	Overrunning signal	A8	Explosion	A9	Fire
A10	Landslide	A11	Diesel leakage	A12	Trapped in door
A13	Water pollution	A14	Train overturning	A15	Train interruption
A16	Train incontrollable				

Additionally, if a single railway accident report described two or more distinct incidents, the incident that directly resulted in economic losses and casualties was selected as the primary event for final analysis. Through this screening criterion, all selected railway accidents were systematically analyzed. Among the 168 investigated railway operational accidents, the dataset comprises 35 derailments, 55 collisions, 12 train sliding incidents, 50 near-misses, 16 overspeed infractions, and 8 dragging accidents, alongside other minor incident types.

### Types of railway accident risk factors

Railway operational accidents are frequently precipitated by the complex interactions of multiple risk factors. Mechanistically, these factors are classified into four primary categories: human, equipment/infrastructural, environmental, and managerial factors. As an accident sequence evolves, these risk factors propagate along causal pathways to form a distinct accident chain. A detailed analysis of these four risk categories within railway operations is presented below:

(1) Human factors: Human factors represent the most active and dynamic elements triggering railway operational accidents. Statistical analysis of the accident reports indicates that human errors primarily manifest as poor communication, driver fatigue, and insufficient skill levels among maintenance personnel, among others. Notably, human factors are implicated in the vast majority of recorded railway accidents.
(2) Equipment factors: This category primarily encompasses the mechanical equipment and physical infrastructure utilized during railway operations. Once technical or mechanical risks materialize, they frequently culminate in catastrophic accidents. Typical examples include braking system failures, settlement or degradation of the track subgrade, and signaling equipment malfunctions.
(3) Environmental factors: Environmental factors are characterized by their capacity to induce sudden emergencies. Because railway lines span vast geographical distances and traverse diverse, complex terrains, they are highly susceptible to abrupt natural phenomena. These factors primarily include torrential rain, severe snowstorms, and rockfalls on the tracks, all of which pose significant safety risks that can directly trigger severe derailments or collisions.
(4) Managerial factors: Managerial factors correlate closely with personnel training, emergency response protocols, and proactive safety prevention. Deficiencies in operational management can lead to inadequate staff skills or incomplete risk assessments, thereby exacerbating the severity of railway emergencies.

Furthermore, to minimize subjective bias and ensure coding consistency, risk factors with semantic similarities are consolidated into unified classifications. For instance, risk factors described in the reports as “inattentive train driver”, “driver distracted” and “driver fatigue” are aggregated under the singular node “driver fatigue.” Additionally, to streamline the topology of the complex network and intuitively illustrate inter-node connections, a unique alphanumeric identifier is assigned to each node. Specifically, nodes H1-H30 represent human factors, N1-N25 denote equipment/infrastructural factors, E1-E11 signify environmental factors, and M1-M12 correspond to managerial factors. Comprehensive details for each coded node are systematically cataloged in Table 2.

**Table 2:** Node information of ROACN.

Node category	Node number	Node	Node number	Node	Node number	Node
**Human factors**	H1	Insufficient skill level of maintenance staff	H2	Improper inspection of maintenance staff	H3	Negligence of maintenance staff
	H4	Improper operation of maintenance staff	H5	Missing or belated of notification by maintenance staff	H6	Inadequate risk estimation of maintenance staff
	H7	Fatigue of signaler	H8	Misjudgment of signaler	H9	Poor communication
	H10	Improper operation of signaler	H11	Inadequate risk estimation of driver	H12	Driver fatigue
	H13	Improper operation of driver	H14	Unused safe speed by driver	H15	Misjudgment of driver
	H16	Hazardous working environment of worker	H17	Misjudgment of worker	H18	Unsafe behavior of worker
	H19	Fatigue of worker	H20	Inadequate risk estimation	H21	Negligence of worker
	H22	Unsafe position of passenger	H23	Misjudgment of passenger	H24	Unsafe behavior of passenger
	H25	Weak safety awareness of passenger	H26	Insufficient skill level of railway staff	H27	Improper inspection of railway staff
	H28	Improper operation of railway staff	H29	Inadequate risk estimation of railway staff	H30	Fatigue of railway staff
**Equipment factors**	N1	Not sending signal	N2	Failure of signal system	N3	Failure of signal equipment
	N4	Failure of braking system	N5	Failure of braking equipment	N6	Component failure or detachment
	N7	Wheels damage	N8	Track deformation or damage	N9	Failure of rack circuit
	N10	Settlement of or damage to track subgrade	N11	Poor wheel and rail adhesion force	N12	Failure of door detection system
	N13	Failure of CCTV	N14	Unfixed of train coupler	N15	Failure of protection equipment
	N16	Poor inspection equipment	N17	Capacitor failure	N18	Brake failure
	N19	Failure of Drainage system	N20	Failure of railway carriage	N21	Degradation of bogies
	N22	Bridge damage	N23	Incomplete design of equipment	N24	Train wheelset damage
	N25	Collapse of retaining wall				
**Environmental factors**	E1	Heavy rain	E2	Strong wind	E3	Snowstorm
	E4	High temperature weather	E5	Wet weather	E6	Flood
	E7	Fog	E8	Fallen leaves on the track	E9	Fallen trees lying on tracks
	E10	Rolling stones on the track	E11	Machine facilities left on the track		
**Management factors**	M1	Insufficient operational supervision	M2	Imperfect management of maintenance and inspection	M3	Imperfect emergency management or organization
	M4	Insufficient safety training of railway staff	M5	Incomplete safety management system	M6	Imperfect management of maintenance practices
	M7	Incomplete assessment and management of risk	M8	Safety management system not operating	M9	Lack of cooperation between departments
	M10	Incomplete safety precautions	M11	Improper work arrangement design	M12	Improper operating standards formulation

### Construction of railway operation accident complex network based on the accident chain

Comprehensive analysis of the accident reports reveals that the risk factors precipitating each incident typically manifest in a chronological sequence. Consequently, the evolutionary process of each accident was expressed in the form of an individual accident chain derived from the textual narratives. These chains were subsequently utilized to construct the directed weighted railway operation accidents complex network. Table 3 illustrates two representative railway accident cases sourced from the RAIB alongside their corresponding extracted accident chains. To ensure systematic extraction, each accident report was evaluated based on critical fields, including the report number, accident location, timestamp, evolutionary progression, direct causes, and underlying systemic causes. By synthesizing this structured information, a cohesive accident description was formulated, from which the constitutive risk factors and sequential accident chains were rigorously identified.

**Table 3:** Two railway accident cases and accident chain extraction in RAIB.

Report number	Accident location	Accident time	Accident description	Risk factor	Accident chain
**R022022**	Carmont, Aberdeenshire	2020-08-12, 09:37	A passenger train (Train 1T08) collided with debris washed from a drain onto the track near Carmont, Aberdeenshire, following very heavy rainfall. Train 1T08 was travelling at 117 km/h, it struck debris washed out from a 15 metre length of steeply sloping drainage trench. The collision caused the train to derail and deviate to the left, before striking a bridge parapet which caused the vehicles to scatter. Three people died, and six people were injured.	N19, E1, E10, M2, M3, A1, A2	E1-M2-N19-E10-M3-A2-A1
**R102018**	north-west Scotland	2018-01-22, 06:03	A passenger train travelling between Mallaig and Fort William in north-west Scotland struck a large landslip on a remote section of line near Glenfinnan. Rainfall and snowmelt together caused landslides, with some gravel falling onto the tracks. Train 1Y42 collided with a landslide while traveling at a speed of approximately 64/h。 The driver of the train applied the brakes approximately 4 seconds before the impact, but was unable to avoid the collision. No one was injured, but some diesel spilled and flowed into the drainage ditch.	E1, A1, A2, E10, M6, M3, N25, A10, A11, A13	E1-A10-M6-N25-E10-M3-A2-A1-A11-A13

**Fig 2:**
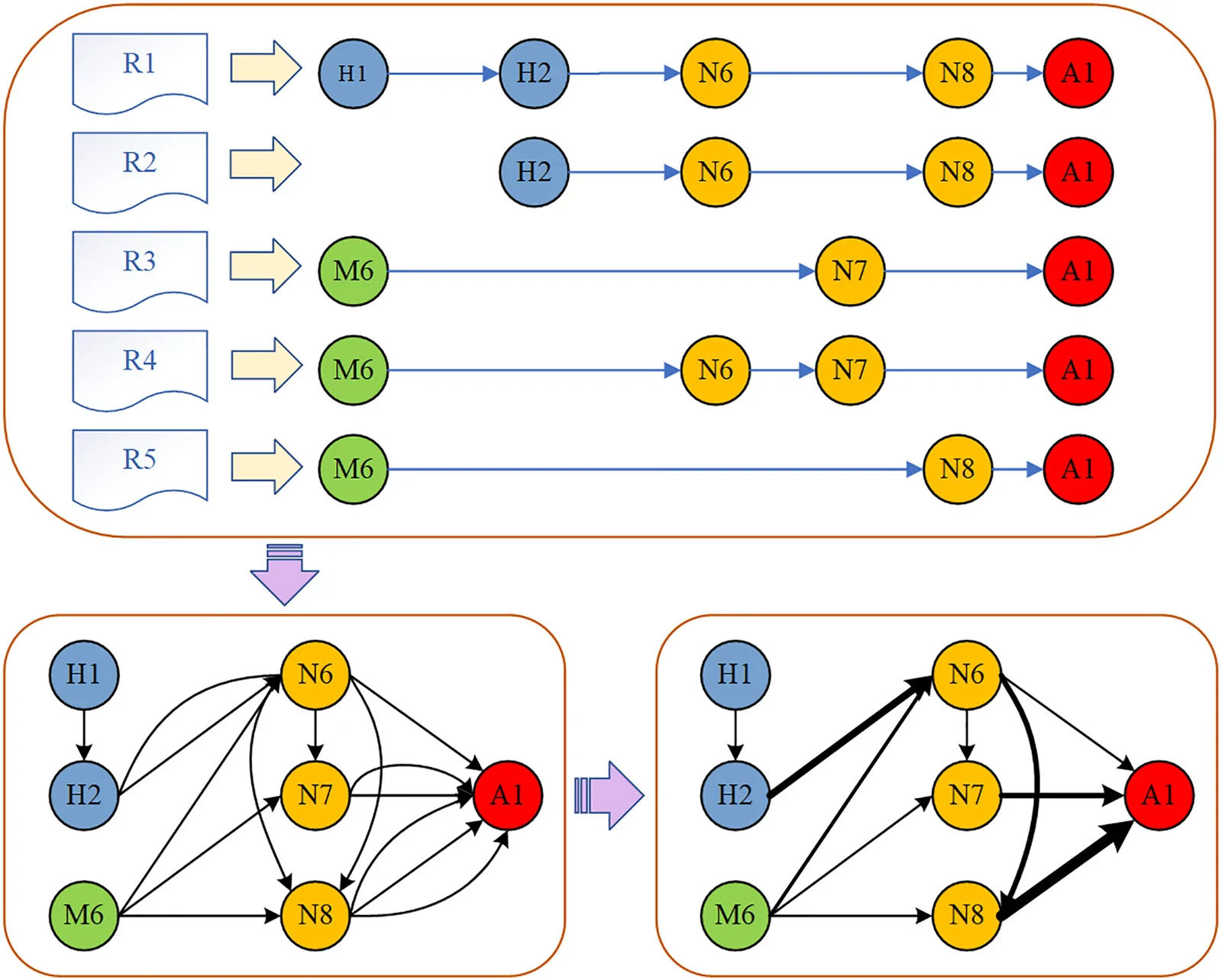
Directed weighted complex network based on accident chain.

Taking railway derailment accidents as an illustrative example, five distinct derailment accident reports were selected to perform the accident chain extraction. Table 4 presents the specific accident chains extracted from these five reports, which collectively illustrate the network modeling approach deployed to develop the ROACN, as depicted in Fig 2. According to the foundational principles of accident chain analysis, each individual chain comprises discrete risk factors and their corresponding causal interrelationships. By extracting the accident chains from these sample derailment reports, 7 distinct risk factors and 15 causal paths were identified. These risk factors and causal paths were subsequently transformed into network nodes and directed edges, respectively, thereby formulating a directed network with overlapping topological paths. Crucially, the co-occurrence frequency of these directed links across the reports was utilized as the edge weight to construct the final directed and weighted complex network.

**Table 4:** Five railway derailment accident reports and accident chains.

Report number	Accident chain
R1	H1-H2-N6-N8-A1
R2	H2-N6-N8-A1
R3	M6-N7-A1
R4	M6-N6-N7-A1
R5	M6-N8-A1

**Fig 3:**
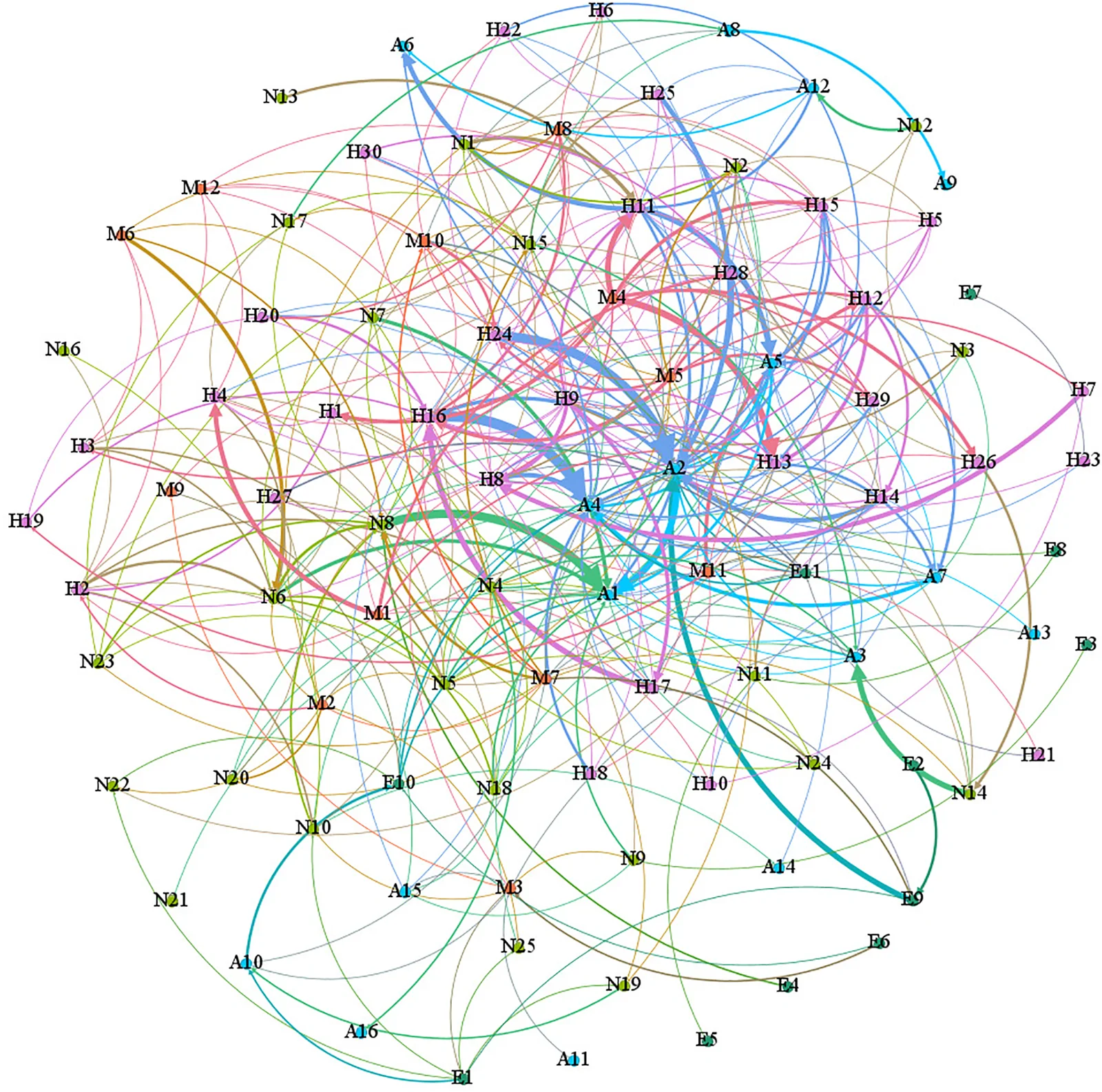
ROACN model based on complex network.

By applying the accident chain method, all 168 railway accident reports were systematically processed, through which the constituent risk factors and their causal interrelationships were comprehensively mapped. Grounded in complex network theory, the global Directed Weighted Railway Operation Accident Complex Network (ROACN) model was successfully constructed. The topological visualization of this network is shown in Fig 3. Dynamically, the ROACN contains 94 distinct nodes and 303 directed edges. Notably, because not all risk factors exhibit direct, localized causal interactions with one another, certain node pairs lack direct edge connections in the ROACN, thereby reflecting the sparse and selective nature of risk propagation pathways within the railway system.

## Results

### Analysis of network structure characteristics

#### Small-world characteristics.

**Fig 4:**
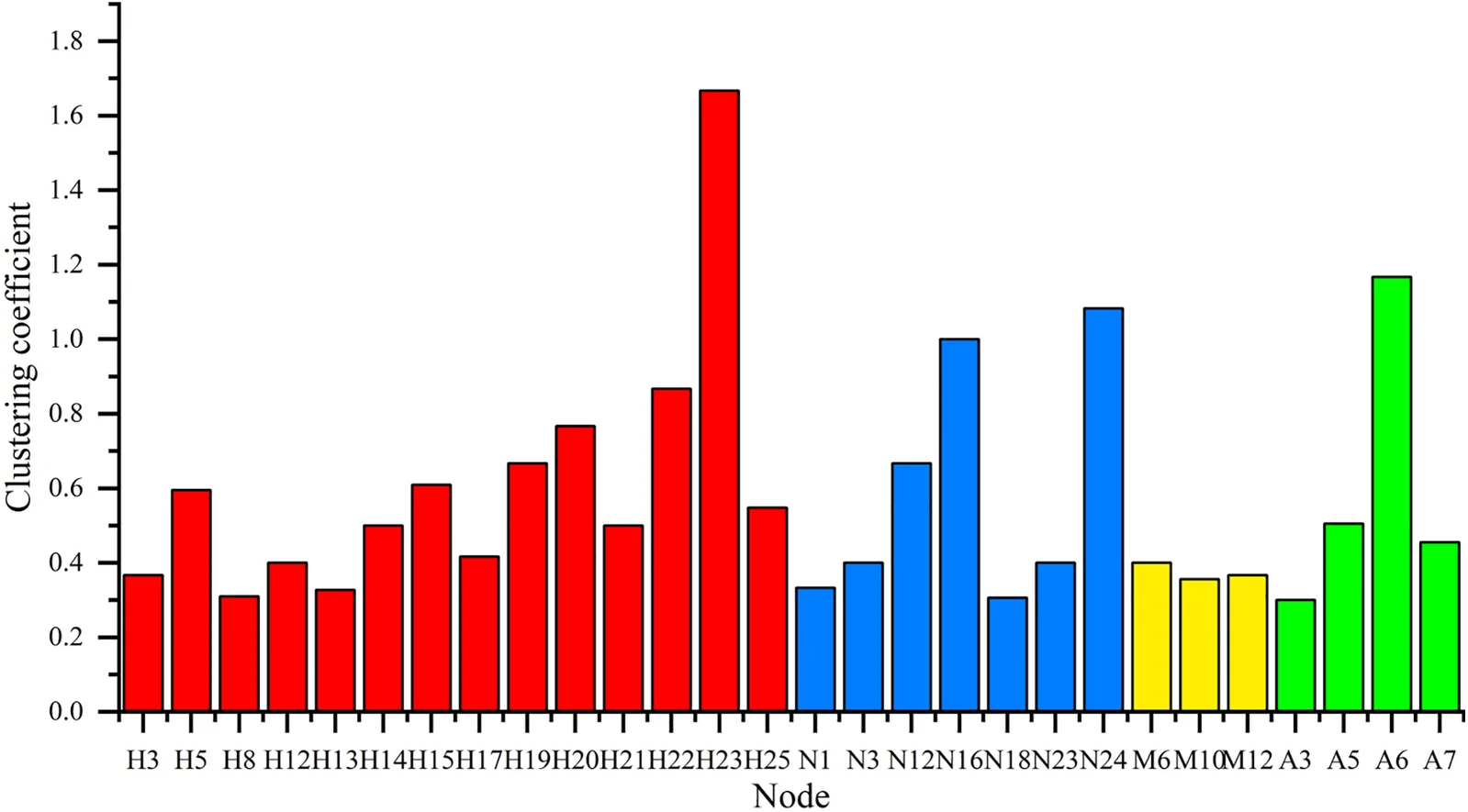
Clustering coefficients of different risk factors in ROACN.

The small-world property of a complex network implies that it exhibits a high clustering coefficient and a short average path length [53]. Within networks possessing small-world topologies, a localized risk propagating from a single node can rapidly diffuse throughout the entire system. For the ROACN, the average path length is calculated to be 3.556. This path length indicates that a specific risk factor can trigger a systemic accident by propagating through an average of approximately three to four intermediary nodes. Furthermore, when taking both edge direction and weight into account, the average weighted clustering coefficient of the ROACN is computed as 0.289, indicating a strong localized clustering tendency among the network nodes. Fig 4 illustrates the clustering coefficients across different risk factors. It is observed that the elements exhibiting high clustering coefficients are predominantly human factors. This phenomenon demonstrates that human factors possess more pronounced clustering characteristics than other risk categories, forming highly cohesive cliques with their neighboring nodes.

Based on the empirical findings above, the ROACN conforms to the criteria of a small-world network. Consequently, when the operational state of a critical node is perturbed, the associated risk can swiftly cascade to adjacent nodes, ultimately precipitating a domino effect. Therefore, accurately identifying and mitigating these critical risk nodes is paramount to promptly severing adverse inter-node correlations and effectively suppressing the escalation of railway accidents.

#### Scale-free characteristics.

**Fig 5:**
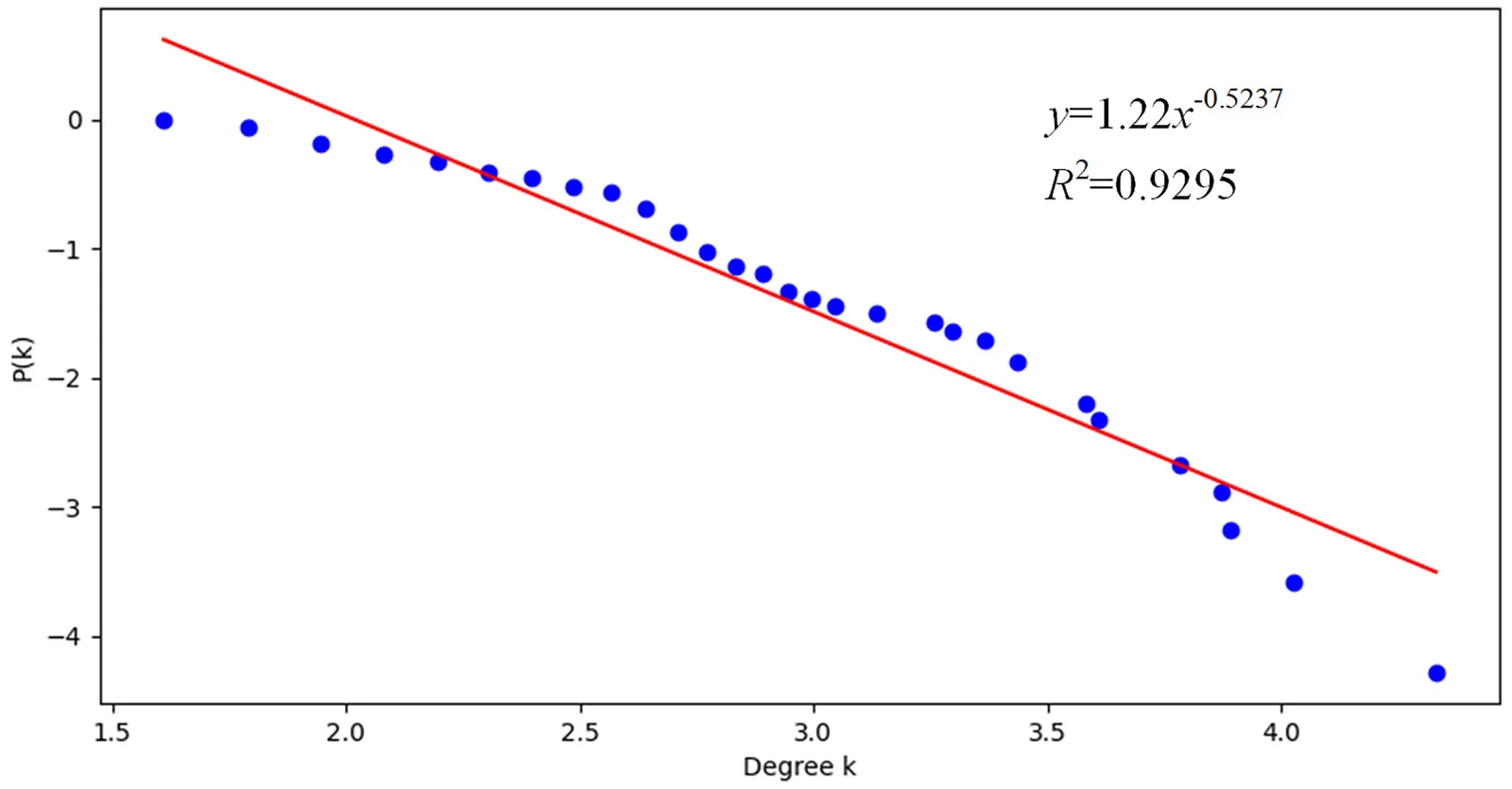
Node accumulation degree Distribution of ROACN.

The scale-free characteristic of a network indicates that a small number of nodes have a large number of connections. Generally, using the cumulative distribution of node degrees to reflect the scale-free characteristics of a network, represented by the equation $P(k)=k^{-\gamma }$. A scatter plot based on node degree values and occurrence frequency is drawn and fitted, as shown in Fig 5. Fig 5 shows that the degree distribution of nodes in ROACN follows a power-law distribution, with a fitting function of $y=\text{1}.\text{22}x^{-\text{0}.\text{5237}}$, $R^{\text{2}}=\text{0}.\text{9295}$. Therefore, ROACN conforms to the characteristics of scale-free networks, where some key nodes are connected to most of the other nodes. By controlling these key nodes, risk propagation can be effectively blocked and accidents can be avoided.

#### Network density.

Network density measures the connectivity between nodes in a network. A higher the network density value indicates a stronger correlation between nodes in the network, leading to greater mutual influence and transmission of risk factors, which can easily trigger railway accidents. The network density of ROACN is calculated to be 0.074. Therefore, the risk evolution path is relatively simple, and the chain reaction caused by risk diffusion is weakened. In addition, by controlling key nodes, the transmission of risks can be blocked and accidents can be prevented.

### Analysis of network node importance indicators

#### Node degree.

**Fig 6:**
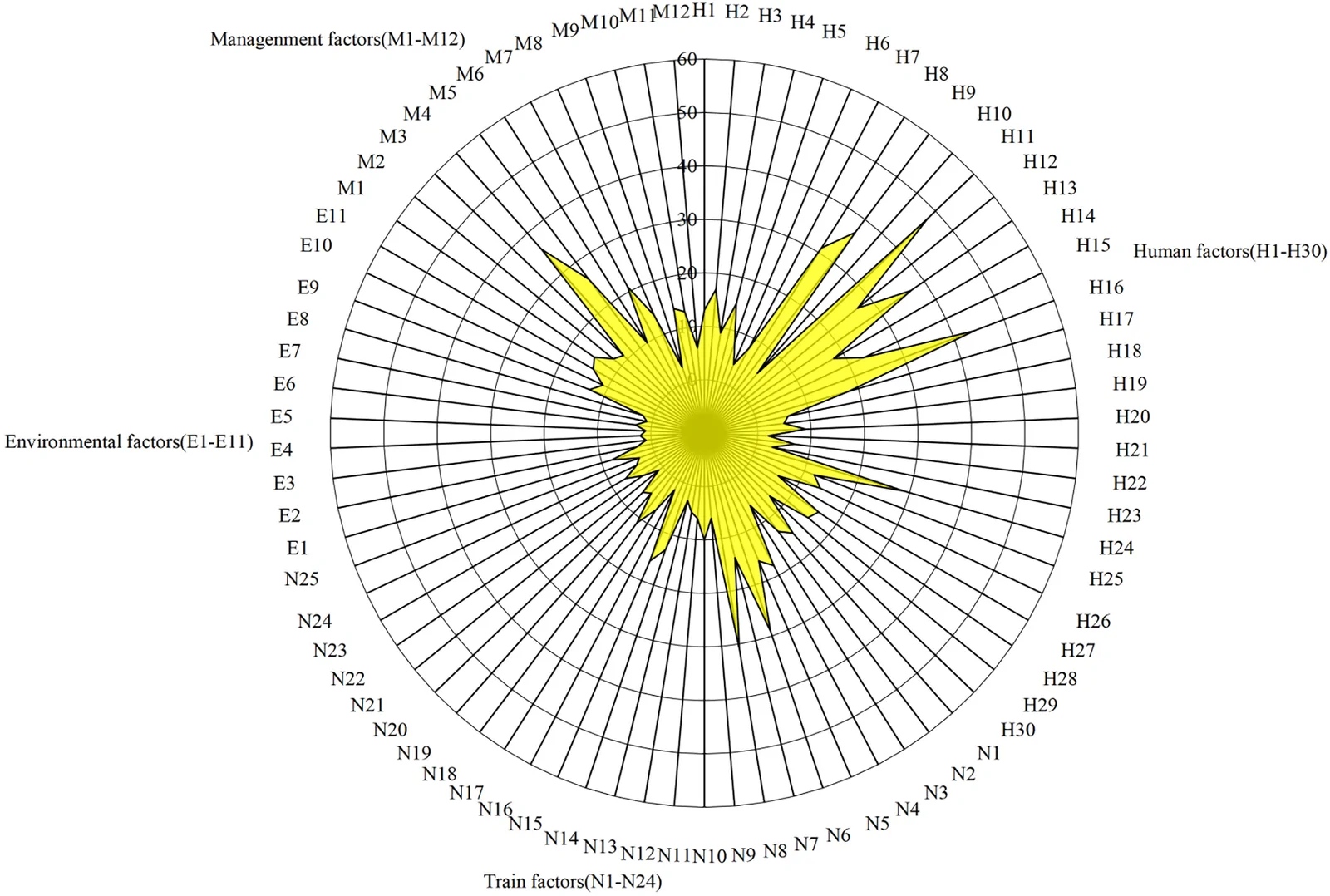
Degree values of different risk factors in ROACN.

Fig 6 displays the degree values of the different nodes within the ROACN. As illustrates in Fig 6, the node degrees in the ROACN are unevenly distributed and exhibit a high degree of topological heterogeneity. When sorted by their respective degree values, the top 10 nodes are H11, H16, H9, H13, M4, H8, N8, H24, N6, and H12. These highly connected vertices function as critical risk hubs within the network, significantly influencing the propagation and materialization of various accidents. When operational risks cascade through nodes with higher degrees, nonlinear coupling and interactive effects with neighboring factors are highly susceptible to occur. This interaction leads to highly volatile and multi-directional pathways for risk propagation, substantially accelerating accident occurrence. Consequently, such dynamics amplify the complexity of subsequent risk mitigation and hinder efforts to truncate risk evolution pathways. Therefore, strategically intervening at network nodes with higher degrees effectively disrupts risk propagation and prevents accidents.

Additionally, the average node degree of the ROACN is 13.426. This topological attribute indicates that each risk factor within the network is, on average, interconnected with more than 13 other risk factors. Mechanistically, this implies that during the evolutionary progression of a railway accident, a state perturbation in a single risk factor possesses the structural capacity to simultaneously alter or cascade into the states of more than 13 neighboring risk factors.

**Fig 7:**
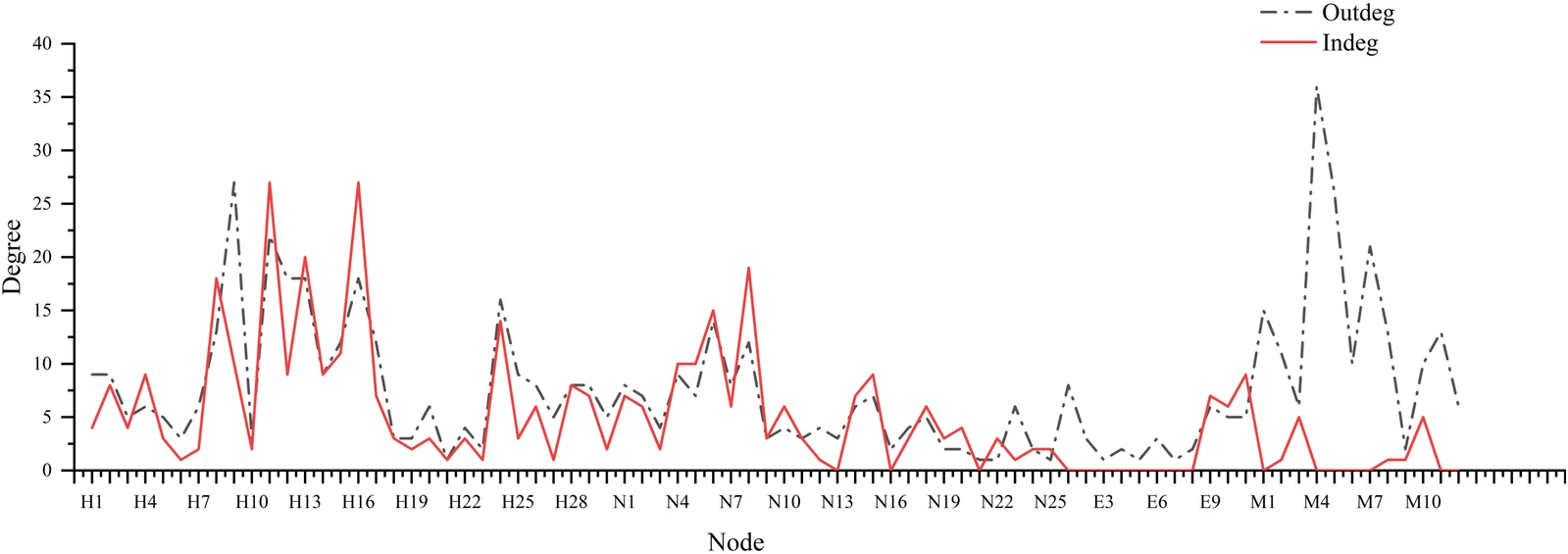
In-degree and out-degree of different risk factors in ROACN.

Because the ROACN operates as a directed network, it is essential to analyze the structural asymmetry between the in-degree and out-degree values of its nodes. A higher node in-degree implies that the corresponding risk factor is more easily triggered or exacerbated by neighboring causal factors. As illustrated in Fig 7, the top 10 nodes with the highest in-degree values are H11, H16, H13, N8, H8, N6, H24, H15, H9, and N5. This distribution reveals that these vulnerable risk factors predominantly consist of human and equipment elements, demonstrating their high susceptibility to external operational disturbances.

Conversely, a higher out-degree value indicates that the node possesses a stronger propensity to trigger or propagate risks to downstream factors. Fig 7 shows that the top 10 nodes with the highest out-degree values are M4, H9, M5, H11, M7, H12, H13, H16, H24, and M1. It is apparent that these highly influential risk factors are concentrated within the human and managerial domains, which highlights their substantial capacity to drive risk evolution and destabilize adjacent network elements.

#### Betweenness centrality.

**Fig 8:**
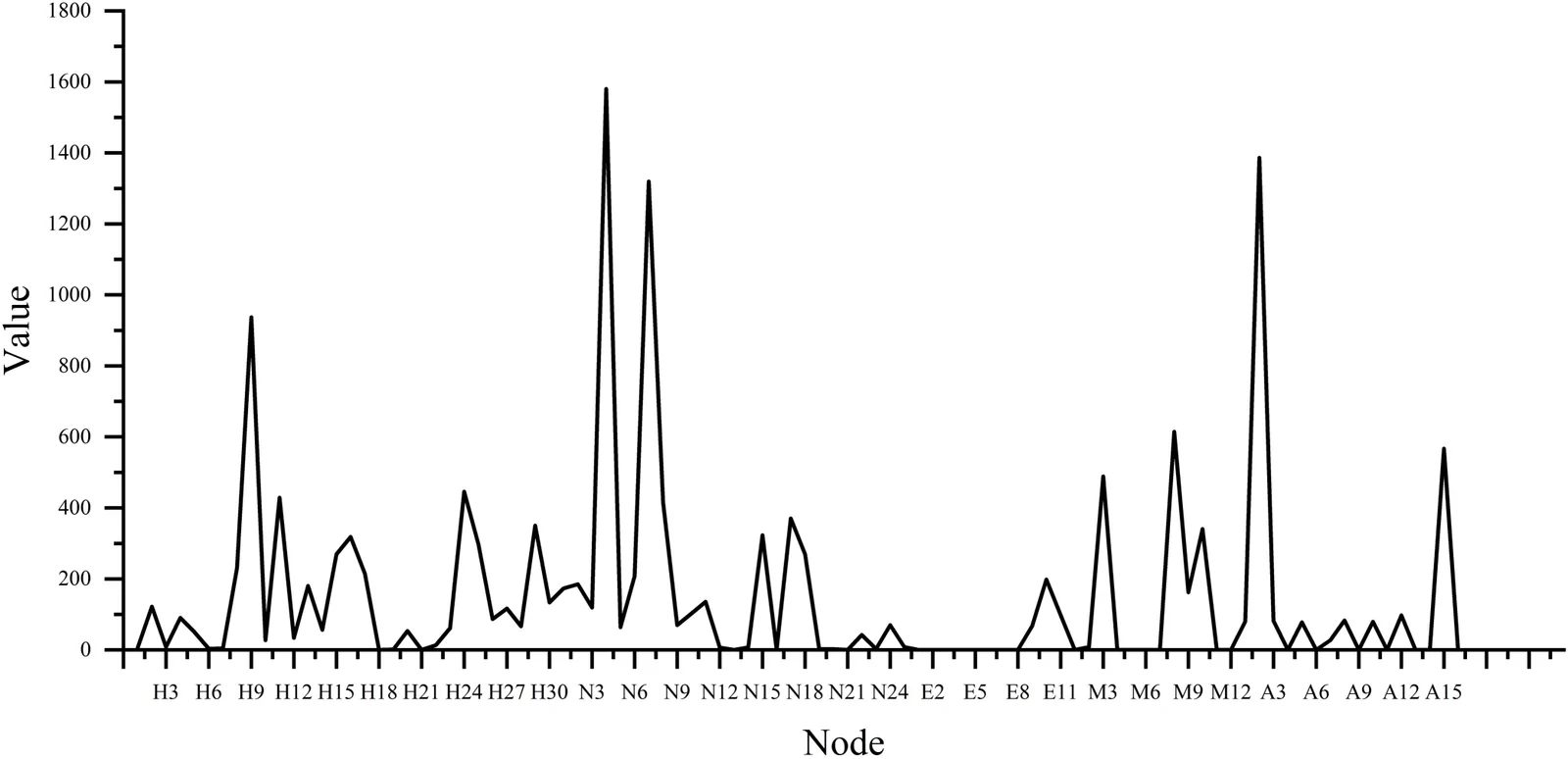
Betweenness centrality of different risk factors in ROACN.

Fig 8 illustrates the betweenness centrality values across all constituent nodes. Nodes exhibiting a betweenness centrality of zero are omitted from the visualization, which indicates that they do not function as vital bridges or intermediaries in the causal interactions among other risk factors. As evidenced by Fig 8, N4 possesses the highest betweenness centrality, demonstrating that a substantial portion of the network’s shortest topological paths traverse this specific element. Consequently, this hub possesses a high structural probability of propagating risks and plays a critical gating role in cascading failures.

Similarly, N7 and H9 display relatively high betweenness centrality values, implying that mechanical defects in train wheels and deficient operational communication serve as crucial bridging conduits that frequently channel risks toward catastrophic railway accidents. Furthermore, key nodes such as M8, M3, H24, H11, N8, N17, and H29 also maintain elevated betweenness centrality profiles, highlights their systemic influence over railway operational safety. From the perspective of accident classifications, A2 exhibits high betweenness centrality, indicating that multiple critical risk paths systematically converge onto these outcomes. This topological convergence suggests that within the current operational risk ecosystem, collision and associated train interruption accidents possess a significantly higher structural likelihood of being triggered relative to other incident types.

#### Closeness centrality.

**Fig 9:**
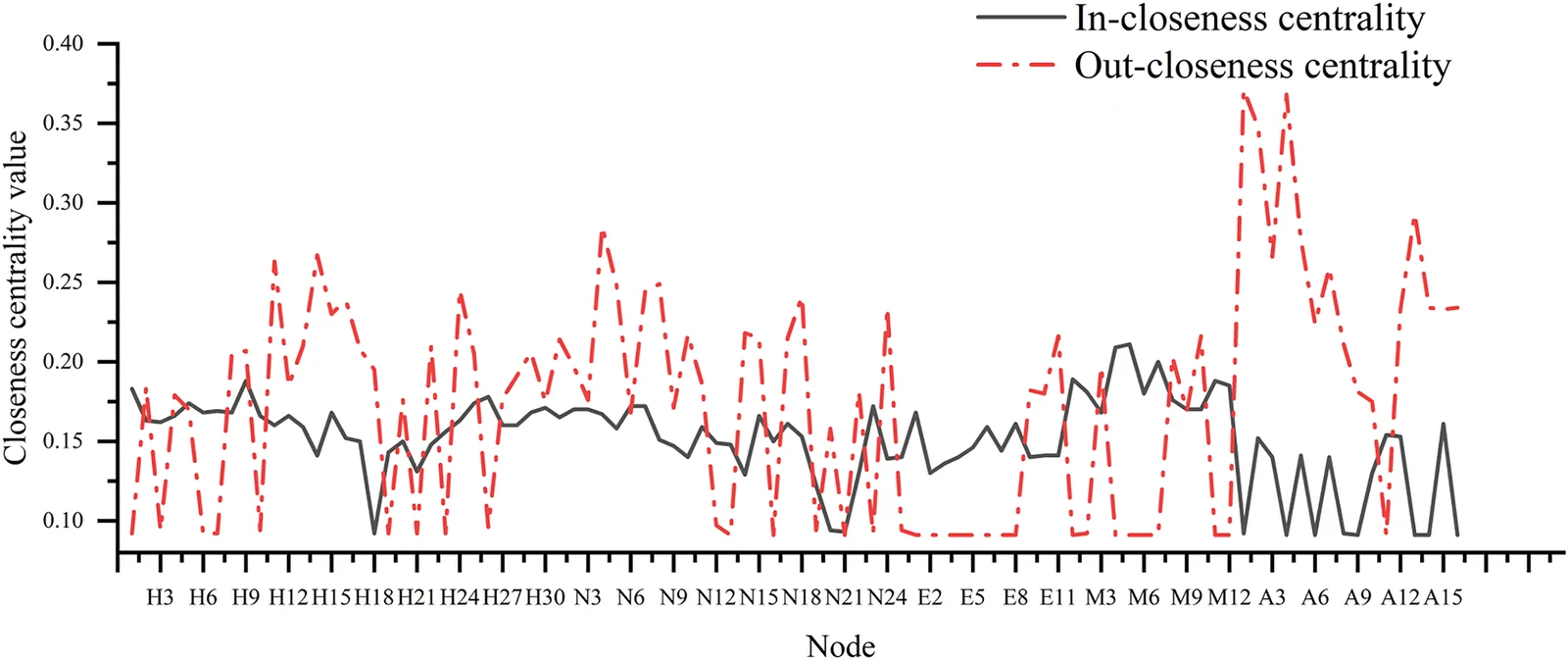
Closeness centrality of different risk factors in ROACN.

Given that the ROACN operates as a directed and weighted network, closeness centrality is decoupled into in-closeness centrality and out-closeness centrality to capture directional proximity. Fig 9 displays the closeness centrality distribution across different risk factors within the ROACN. When sorting the nodes by their in-closeness centrality values, the top 10 positions are occupied by N4, H14, H11, N8, N5, H24, N7, N18, H16, and N24. This distribution reveals that these specific nodes possess a high structural capacity to establish rapid, short-path connections from other risk sources. Additionally, A1, A2, and A4 exhibit elevated in-closeness centrality profiles, demonstrating that these three accident types are highly susceptible to being triggered by cumulative upstream network perturbations.

Conversely, when evaluated based on out-closeness centrality values, the top 10 nodes are M5, M4, M7, M1, M11, H9, M12, H1, M2, and M6. It is evident that managerial factors dominate the high out-closeness spectrum, reflecting their exceptional propagation efficiency and powerful outward influence over downstream network nodes. Furthermore, specific terminal events, including A2, A11, and A12 that display significantly higher out-closeness centrality. This topology suggests that these three operational events possess a stronger propensity to spill over and adversely affect subsequent network elements.

#### Eigenvector centrality.

**Fig 10:**
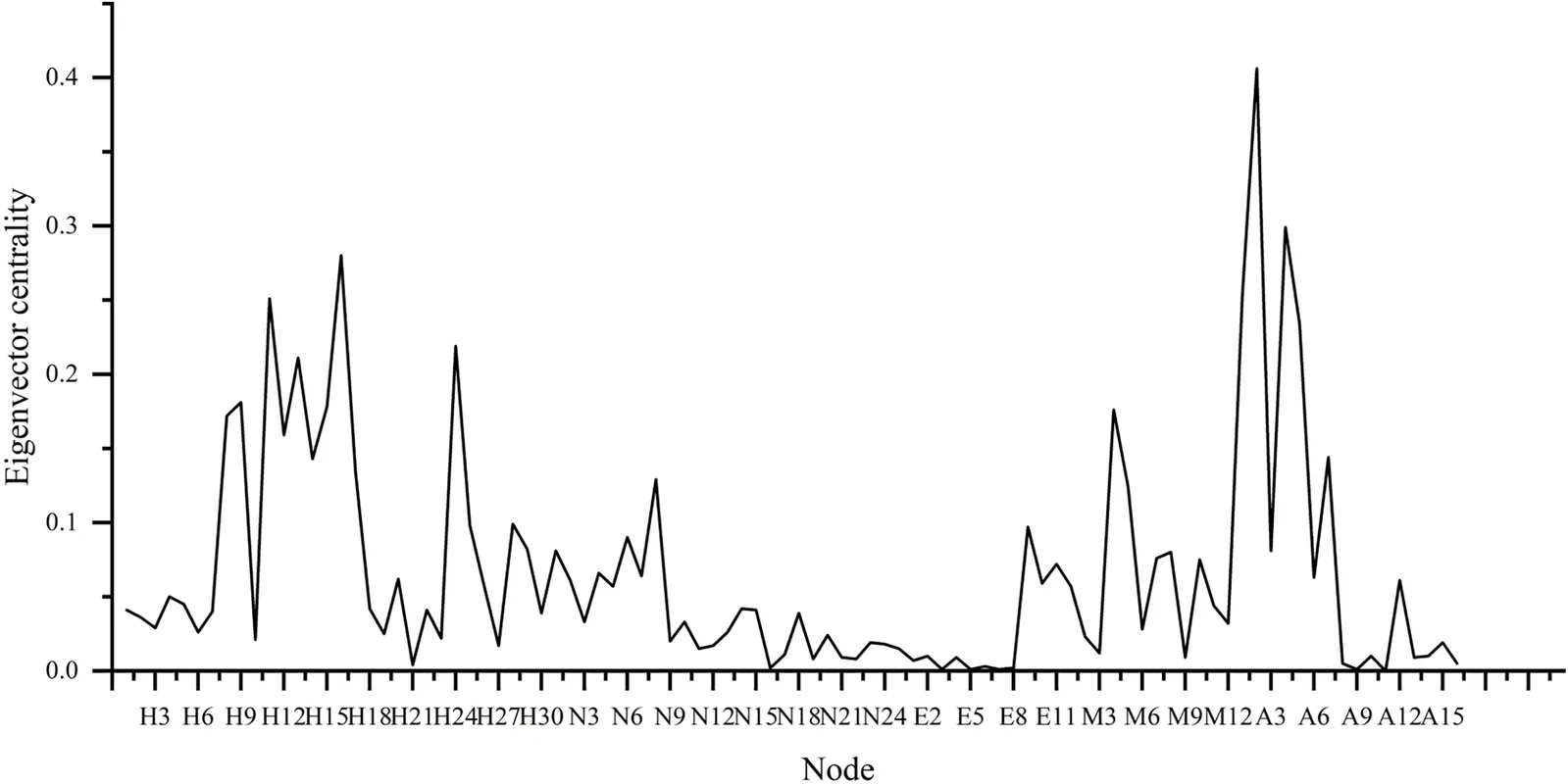
Eigenvector centrality of different risk factors in ROACN.

Fig 10 illustrates the eigenvector centrality values for the various risk factors within the ROACN. When sorting the nodes by their respective eigenvector centrality values, the top 10 positions are occupied by H16, H11, H24, H13, H9, H15, M4, H8, H12, and H14. Strikingly, among these top 10 critical factors, nine belong to the human factors category. This disproportionate distribution indicates that human risk factors exhibit a significantly higher affinity for interconnecting with other highly influential network hubs compared to remaining factor types. Specifically, node $H16$ possesses the highest eigenvector centrality, demonstrating that its adjacent topological neighbors are themselves of paramount systemic importance and function as major risk conduits within the railway operational ecosystem.

#### Structural hole.

**Fig 11:**
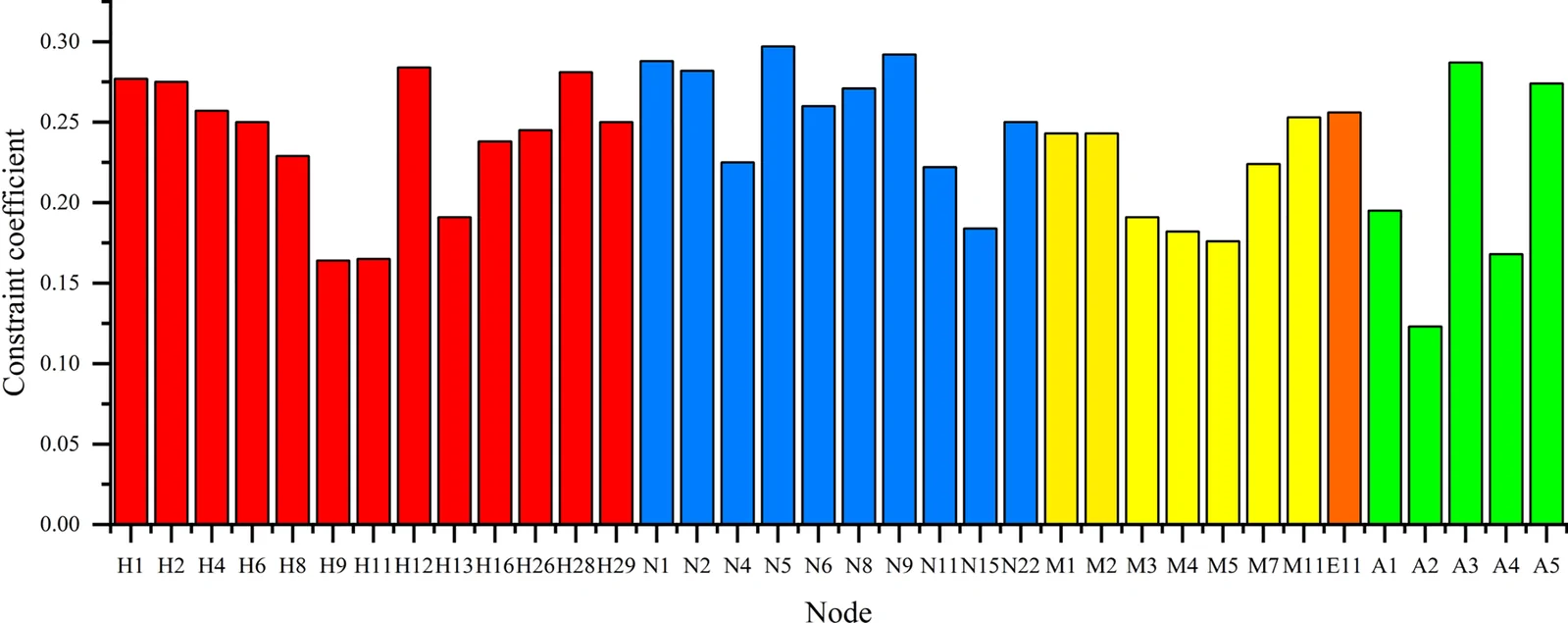
Structural hole of different risk factors in ROACN.

Fig 11 illustrates the structural hole profiles quantified by the constraint coefficients for the various risk factors within the ROACN. It is evident that nodes exhibiting lower constraint coefficient values are predominantly concentrated within the human and managerial domains. Among all investigated risk factors, H9 possesses the minimum constraint value of 0.164, ranking first globally, while H11, M5, M4, and N15 occupy the second through fifth positions, respectively. These elements with relatively low constraint coefficients hold strategic brokerage positions within the railway accident causation network, serving as crucial topological bridges that span otherwise disconnected clusters. Mechanistically, when operational abnormalities or perturbations occur at these structural hole sites, risks can diffuse rapidly and unimpeded throughout the entire system. Consequently, implementing proactive and targeted interventions to control these low-constraint nodes effectively truncates rapid risk dissemination pathways, thereby substantially bolstering the dynamic resilience and systemic safety of railway operations.

### Result of node comprehensive importance

The individual topological investigations presented above yield diverse importance profiles across different evaluation indicators. However, determining the critical risk factors of railway accidents based solely on a single topological metric lacks scientific comprehensiveness. Consequently, it is necessary to simultaneously account for the systemic impacts of different evaluation indicators by integrating the respective mathematical advantages of various node evaluation dimensions. To address this requirement, this study introduces a comprehensive node importance evaluation framework developed through a combinatorial weighting-TOPSIS methodology.

**Fig 12:**
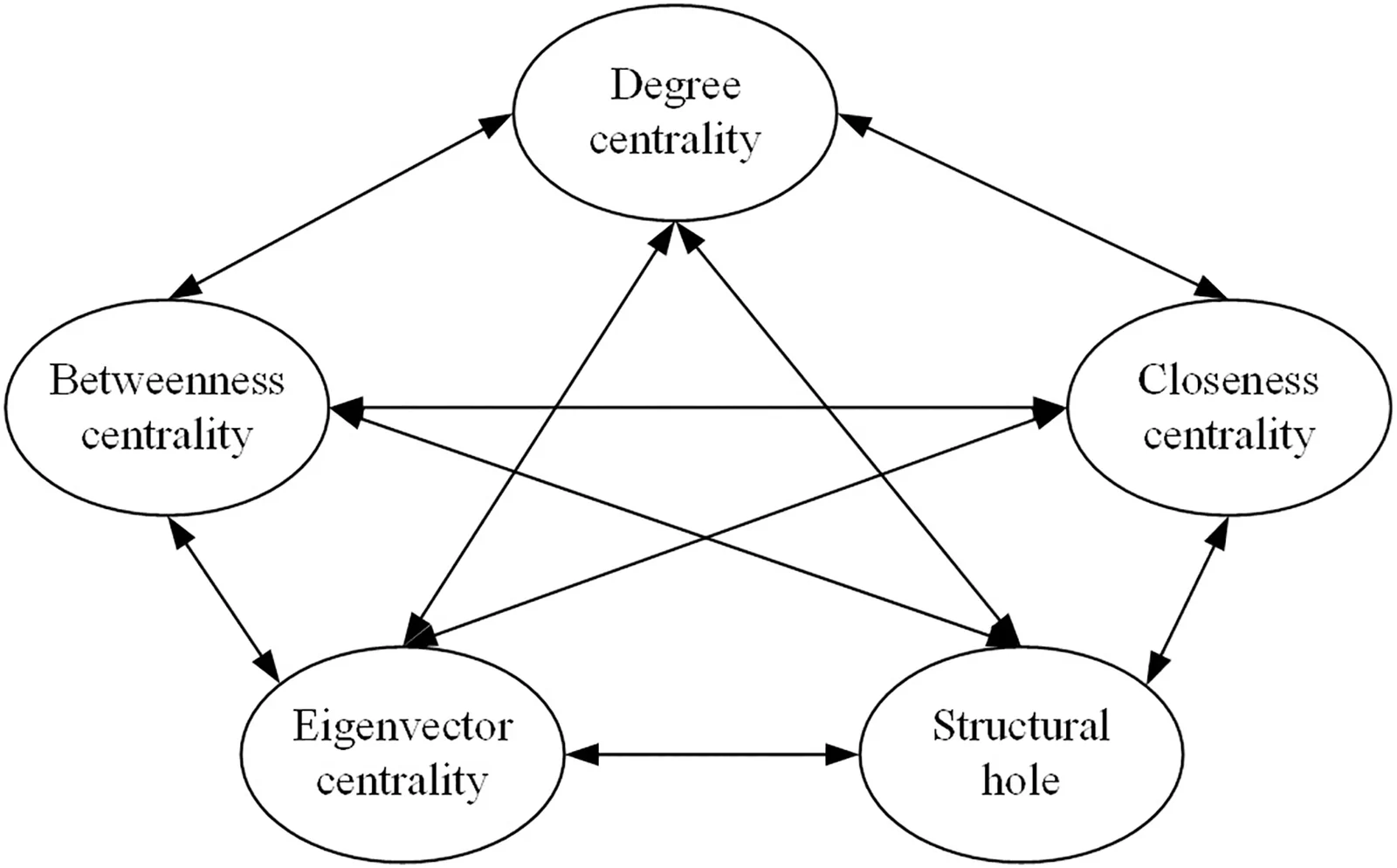
Network hierarchy model of different indicators.

Mechanistically, the preprocessing of the raw topological indicator data from the ROACN yields a normalized decision matrix. Following this standardization, the CRITIC method establishes the objective weights for each structural indicator, which are systematically cataloged in Table 7. Concurrently, the ANP method determines the subjective weights of each indicator. To accommodate the intricate mutual dependencies and feedback loops among the various topological metrics, a rigorous network hierarchy model of the different evaluation indicators is constructed, as illustrated in Fig 12.

Utilizing a standard 1–9 scaling metric, five experts evaluate the relative importance of each topological indicator against the others to construct the initial pairwise comparison matrices. Table 5 illustrates a representative comparison matrix evaluated by one expert regarding the relative importance of node degree against alternative indicators. To mitigate subjective variances among different evaluators, the individual assessments undergo an aggregation process wherein the mathematical average of all expert responses formulates the final consensus comparison matrix. A rigorous consistency check is executed on this final matrix, yielding a consistency ratio of CR=0.086<0.1, which indicates satisfactory consistency in the pairwise judgments. Subsequently, the normalized eigenvectors corresponding to the maximum eigenvalues of the different indicators are integrated to construct the unweighted supermatrix. This unweighted supermatrix is further normalized to generate the weighted supermatrix, which ultimately converges into the limit supermatrix through continuous power iterations, as systematically detailed in Table 6.

**Table 5:** Comparison matrix of the importance of node degree to other indicators.

	BC	DC	EC	CC	SH
**BC**	1	9	8	7	7
**DC**	1/9	1	1/2	1/7	1/7
**EC**	1/8	2	1	1/4	1/4
**CC**	1/7	7	4	1	1/2
**SH**	1/7	7	4	2	1

**Table 6:** Limit hypermatrix.

	BC	${𝐃}_{𝐢𝐧}$	${𝐃}_{𝐨𝐮𝐭}$	${𝐂𝐂}_{𝐢𝐧}$	${𝐂𝐂}_{𝐨𝐮𝐭}$	EC	SH
**BC**	0.568207	0.568207	0.568207	0.568207	0.568207	0.568207	0.568207
${𝐃}_{𝐢𝐧}$	0.011505	0.011505	0.011505	0.011505	0.011505	0.011505	0.011505
${𝐃}_{𝐨𝐮𝐭}$	0.034852	0.034852	0.034852	0.034852	0.034852	0.034852	0.034852
${𝐂𝐂}_{𝐢𝐧}$	0.077464	0.077464	0.077464	0.077464	0.077464	0.077464	0.077464
${𝐂𝐂}_{𝐨𝐮𝐭}$	0.104356	0.104356	0.104356	0.104356	0.104356	0.104356	0.104356
**EC**	0.069902	0.069902	0.069902	0.069902	0.069902	0.069902	0.069902
**SH**	0.133713	0.133713	0.133713	0.133713	0.133713	0.133713	0.133713

Table 7 provides the final subjective weights, objective weights, and comprehensive weights compiled for each individual indicator. Based on these comprehensive weight profiles, the TOPSIS framework determines the comprehensive importance scores for all constituent nodes within the ROACN, with the final rankings systematically presented in Table 8. By evaluating the multifaceted attributes of degree centrality, betweenness centrality, closeness centrality, eigenvector centrality, and structural hole constraints simultaneously, the top 10 nodes of global importance are identified as N4, N7, H9, H11, M8, H24, H16, N8, M3, and H15.

**Table 7:** Comprehensive weights of various indicators.

Indicators	Subjective weight	Objective weight	Comprehensive weight
BC	0.568207	0.219581	0.4670
${\text{D}}_{\text{in}}$	0.011505	0.122418	0.0437
${\text{D}}_{\text{out}}$	0.034852	0.108188	0.0561
${\text{CC}}_{\text{in}}$	0.077464	0.157840	0.1008
${\text{CC}}_{\text{out}}$	0.104356	0.106971	0.1051
EC	0.069902	0.121534	0.0849
SH	0.133713	0.163466	0.1424

**Table 8:** The nodes importance ranking results of risk factors in the ROACN based on TOPSIS.

Number	Node	${𝐃}_{𝐢}^{+}$	${𝐃}_{𝐢}^{-}$	${𝐙}_{𝐢}$	Rank
N4	Failure of braking system	0.284502	0.902583	0.760336	1
N7	Wheels damage	0.324265	0.76714	0.702892	2
H9	Poor communication	0.355303	0.69177	0.66067	3
H11	Inadequate risk estimation of driver	0.572814	0.591697	0.508108	4
M8	Safety management system not operating	0.54699	0.521069	0.487866	5
H24	Unsafe behavior of passenger	0.57646	0.53535	0.481512	6
H16	Hazardous working environment of worker	0.633942	0.549577	0.464358	7
N8	Track deformation or damage	0.613142	0.501543	0.449942	8
M3	Imperfect emergency management	0.630995	0.498959	0.441574	9
H15	Misjudgment of driver	0.665191	0.484405	0.42137	10
…	…	…	…	…	…
N16	Poor inspection equipment	0.95926	0.157303	0.140882	76
H21	Negligence of worker	0.942235	0.131938	0.122827	77
N21	Degradation of bogies	0.960343	0.116366	0.108076	78

Concurrently, Table 9 displays the evaluation results regarding the comprehensive importance of specific railway accident classifications. The ranking indicates that the top 5 accident types possessing the highest risk propagation importance are A2, A15, A5, A1, and A4. These numerical outcomes obtained from the multi-attribute calculation closely align with empirical industry benchmarks and historical data. From both systemic and practical perspectives, these alignments verify that the proposed comprehensive node importance evaluation methodology operates with high accuracy and provides a reliable framework for risk identification.

**Table 9:** The nodes importance ranking results of accident types in the ROACN based on TOPSIS.

Number	Node	${𝐃}_{𝐢}^{+}$	${𝐃}_{𝐢}^{-}$	${𝐙}_{𝐢}$	Rank
A2	Collision	0.047307	0.980815	0.953987	1
A15	Train interruption	0.569894	0.533577	0.483544	2
A5	Overspeed	0.738422	0.489674	0.398726	3
A1	Derailment	0.803027	0.47489	0.371612	4
A4	Near miss	0.844011	0.491833	0.368182	5
…	…	…	…	…	…
A6	Drag	0.941229	0.147958	0.135843	14
A16	Train incontrollable	0.968464	0.142142	0.127986	15
A9	Fire	0.977785	0.089545	0.083896	16

### Robustness analysis of ROACN

#### Evaluation metrics for network robustness.

Network Efficiency (NE) operates as a crucial topological metric for quantifying the transmission efficiency of risk or information within a complex network structure. When a network is subjected to continuous targeted or random attacks, the elongation of the shortest path lengths among surviving nodes leads to a systematic decline in risk transmission efficiency. Mathematically, network efficiency is calculated as follows:


$$\begin{array}{c} NE=\frac{\text{1}}{N(N-\text{1})}\sum_{i\neq j}\frac{\text{1}}{l_{ij}} \end{array}$$ (44)

where $l_{ij}$ represents the shortest path length between any two nodes i and j in the network. When EF=1, it indicates that all nodes in the entire network are fully connected, while EF=0 means that all nodes in the network are isolated.

The Relative Size of the largest connected component (RS) refers to the specific subnetwork that encompasses the maximum number of interconnected nodes after a perturbation. When the network is subjected to attacks, it may be partitioned into two or more subnetworks. The existence of these subnetworks implies that risks may propagate through the network structure, thereby affecting a larger range of nodes. The relative size of the largest connected component is calculated as follows:


$$\begin{array}{c} \begin{array}{c} RS=\frac{N^{\prime }}{N} \end{array} \end{array}$$ (45)

where $N^{\prime }$ represents the number of nodes in the largest connected component. N represents the total number of nodes in the initial network.

#### Attack strategies.

The robustness of the ROACN is systematically evaluated under two distinct operational disruption paradigms: random failure attacks and intentional targeted attacks. The random failure attack strategy treats all network components indiscriminately, meaning that the activation probability of an attack remains uniform across all nodes, and individual failure events possess no statistical correlation. Conversely, intentional targeted attacks involve the purposeful selection of specific nodes based on the underlying topological structure of the network to identify and disrupt vertices with higher structural prominence. These targeted interventions typically exhibit cumulative and continuous degradation characteristics. In this simulation experiment, the targeted attack framework encompasses strategies derived from node degree centrality, betweenness centrality, closeness centrality, eigenvector centrality, structural hole, and the comprehensive node importance proposed in this study. The operational definitions of these seven competitive attack strategies are cataloged below:

(1) Random attack (RA): Randomly select nodes in the network for attack.
(2) Degree centrality attack (DCA): Attack nodes in ROACN based on their degree centrality importance.
(3) Betweenness centrality attack (BCA): Attack nodes in ROACN based on their betweenness centrality importance.
(4) Closeness centrality attack (CCA): Attack nodes in ROACN based on their closeness centrality importance.
(5) Eigenvector centrality attack (ECA): Attack nodes in ROACN based on their eigenvector centrality importance.
(6) Structural hole attack (SHA): Attack nodes in ROACN based on their structural hole importance.
(7) Comprehensive importance attack (CIA): Attack nodes in ROACN based on their comprehensive importance.

### Analysis of simulation results

To systematically evaluate and compare the disruptive effectiveness of the seven distinct node attack strategies, network efficiency (NE) and the relative size of the largest connected component (RS) are monitored under each paradigm. This comparative framework isolates the respective impacts of RA, DCA, BCA, CCA, ECA, SHA, and CIA on the structural robustness of the ROACN. Within the topological context of the ROACN, where each node typifies a specific operational risk or accident event, sequential node removal progressively depletes the active vertices within the topology. This continuous dismantling inevitably suppresses the overall transmission efficiency of risk cascades across the remaining network structure, manifesting as a monotonic decline in network efficiency.

#### Robustness analysis of static network node failure.

**Fig 13:**
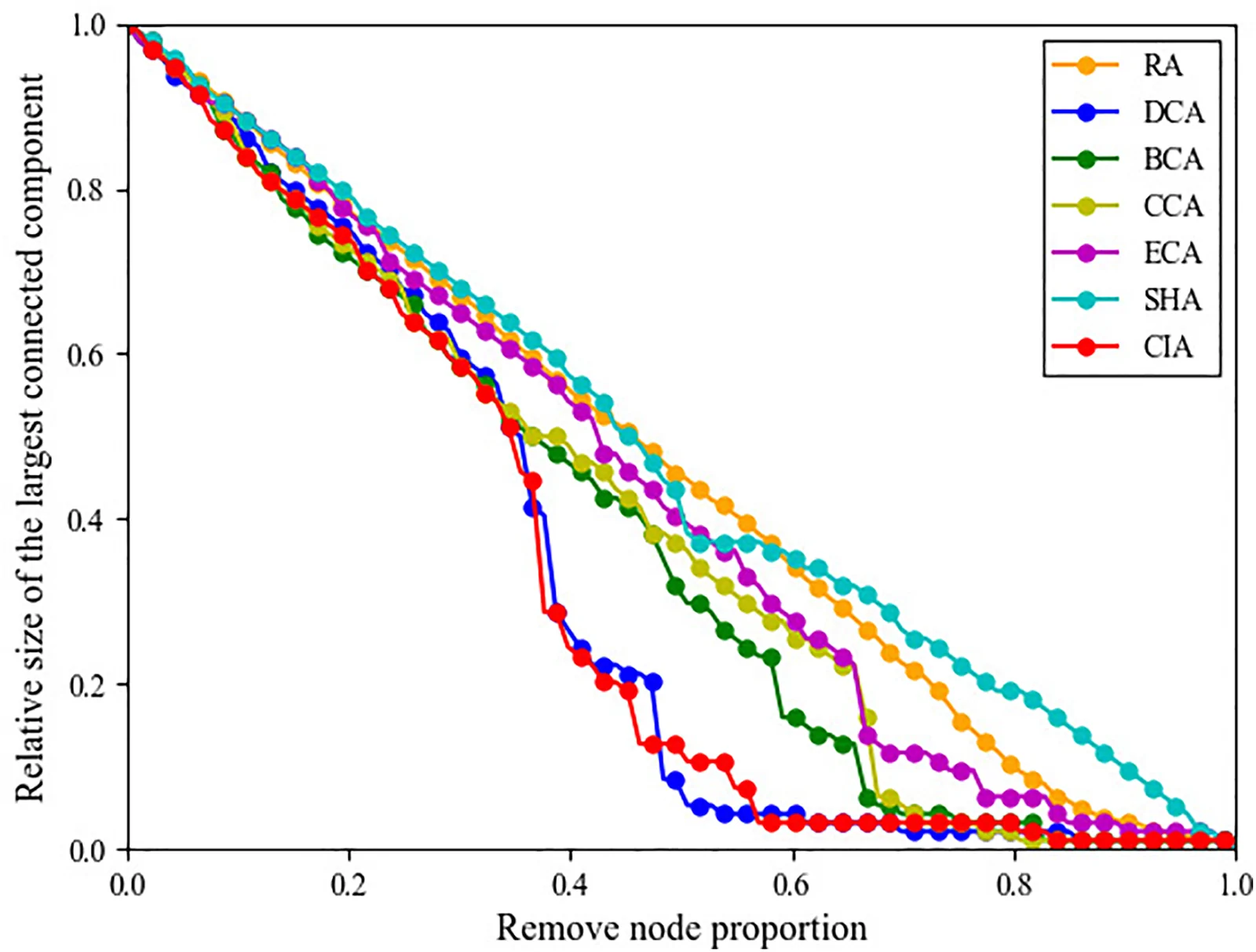
The static change of the relative size of the largest connected component under different attacks.

**Fig 14:**
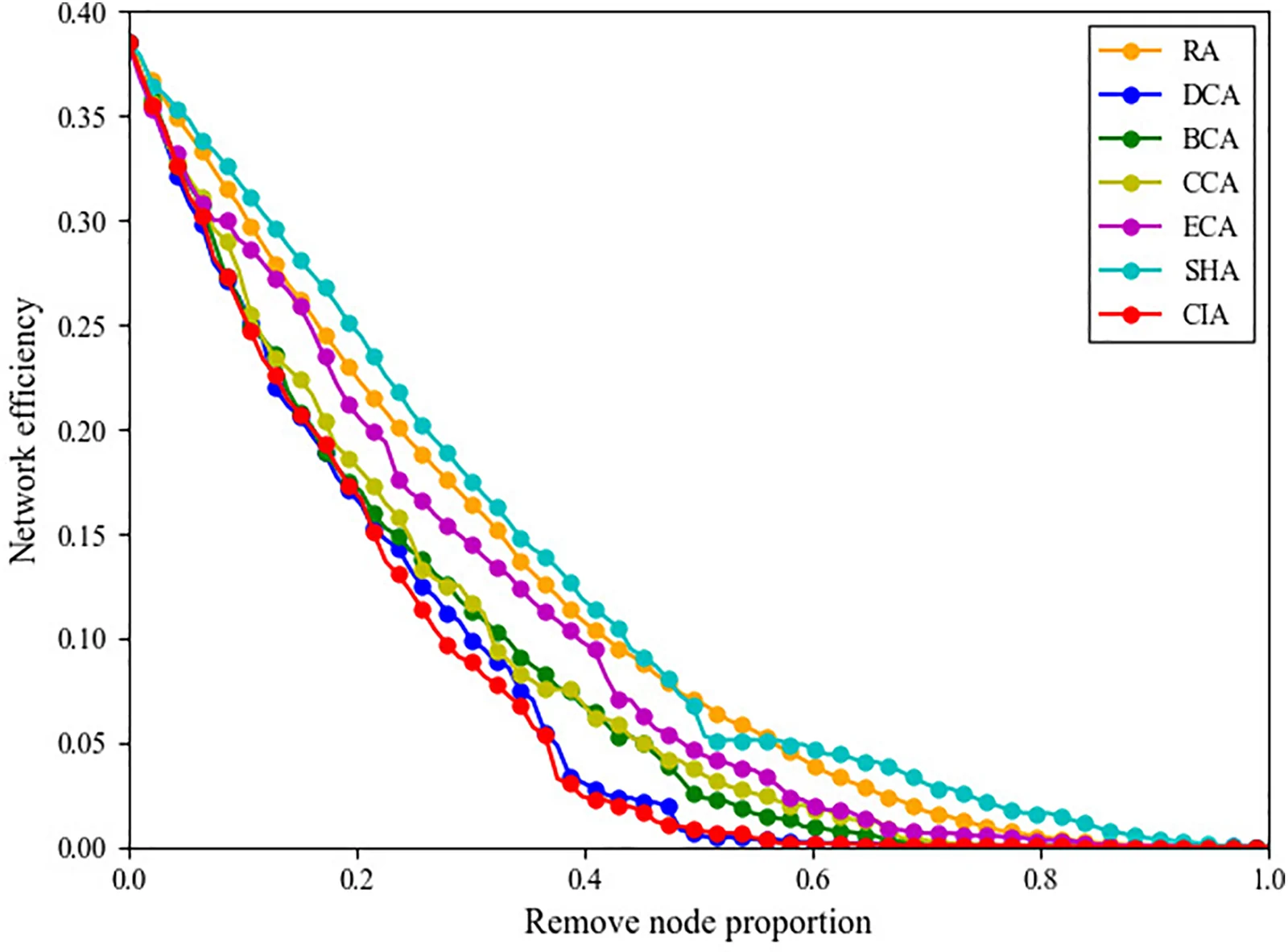
The static change of network efficiency under different attacks.

The evaluation of network degradation under the competitive attack strategies of RA, DCA, BCA, CCA, ECA, SHA, and CIA on the initial network configuration constitutes the static network robustness analysis. Under this static evaluation framework, the initial simulation step targets and disables the top 10% of nodes according to their static importance rankings under each specific strategy. Following this intervention, the corresponding variations in the network robustness indicators are computed for the initial attack iteration. Subsequently, the next simulation step targets an expanded cumulative fraction of nodes by increasing the node removal ratio β based on the baseline rankings, which triggers a recalculation of the topological indicators. This iterative elimination progresses sequentially until all nodes within the network topology experience complete structural failure, where both the relative size of the largest connected component and network efficiency drop precisely to 0, signaling the formal termination of the simulation. Fig 13 displays the static variation of the relative size of the largest connected component under the different attack strategies, while Fig 14 illustrates the corresponding static response of network efficiency.

As displayed in Fig 13, the baseline RS value of the undisturbed network is normalized to 1. Upon the execution of the various attack strategies, the RS profiles of the ROACN decrease monotonically as the node removal ratio β escalates. Based on the degradation trajectory of the RS curves, the overall destructive capacity of the attack strategies ranks in the descending order of CIA > DCA > BCA > CCA > ECA > RA > SHA.

Specifically, comparing different intentional attack strategies, when the node removal ratio β<0.25, CIA, DCA, BCA, CCA, and ECA show similar attack effects compared to RA and SHA, and the relative size of the largest connected component decreases rapidly. When the node removal ratio β≥0.25, the curves of CIA and DCA change rapidly, showing excellent attack effects. When the node removal ratio β≥0.6, the curves of BCA, CCA, and ECA show a rapid decrease trend. When the node removal ratio β=0.83, the RS value of the CIA strategy is 0, indicating network collapse. This indicates that when ROACN is subjected to random failure attacks, the network exhibits strong robustness, while under intentional attacks, the stability and robustness of the network are poor, especially under CIA and DCA attacks, where the network structure changes rapidly, and its functionality decreases sharply, approaching collapse.

Fig 14 illustrates the static response of NE under the seven competitive operational disruption paradigms. As displayed in Fig 14, the initial baseline NE of the undisturbed topology is 0.3848. Upon the deployment of the different attack strategies, the overall NE values decrease gradually as the node removal ratio β escalates. Additionally, compared to the benign trajectory of RA, the remaining six intentional targeted attack strategies exhibit significantly higher degradation rates. Particularly, under the CIA framework, the network efficiency plummets most rapidly among all tested models, reaching an absolute value of 0 when the removal ratio reaches β=0.58. Based on the structural degree of decrease in NE, the destructive capacity of these strategies ranks in the descending order of CIA > DCA > BCA > CCA > ECA > RA > SHA.

Specifically, the CIA strategy and DCA strategy have the greatest impact on network efficiency when launching intentional attacks on the network; under these two attack strategies, when the node removal ratio β=0.6, the network efficiency is 0, indicating network collapse. When the node removal ratio β=0.4, the trend of network efficiency changes slows down for different attack strategies. Throughout the process of node removal, the CIA strategy consistently exhibits the fastest decline in global network efficiency, followed by the DCA strategy. This indicates that the efficiency robustness of the network is weaker under these two attack strategies.

#### Robustness analysis of dynamic network node failure.

The dynamic network robustness analysis differs inherently from the static approach because it focuses on the reconstructed network topology following each sequential node entry as the newly updated analytical object. Specifically, on the initial network configuration G, the competitive attack strategies of RA, DCA, BCA, CCA, ECA, SHA, and CIA are systematically deployed. The top 10% of nodes according to the real-time importance rankings under the different attack strategies are selected for the i−th node attack iteration, which yields the modified network $G_{i}$ after the attack. The remaining topology $G_{i}$ then undergoes an immediate recalculation for its degree centrality, betweenness centrality, closeness centrality, eigenvector centrality, structural hole constraint, and comprehensive importance values. This iterative recalculation formulates dynamically updated targeted attack sequences. Based on the adjusted configuration of $G_{i}$, these new attack strategies are applied for the subsequent (i+1)−th attack iteration.

**Fig 15:**
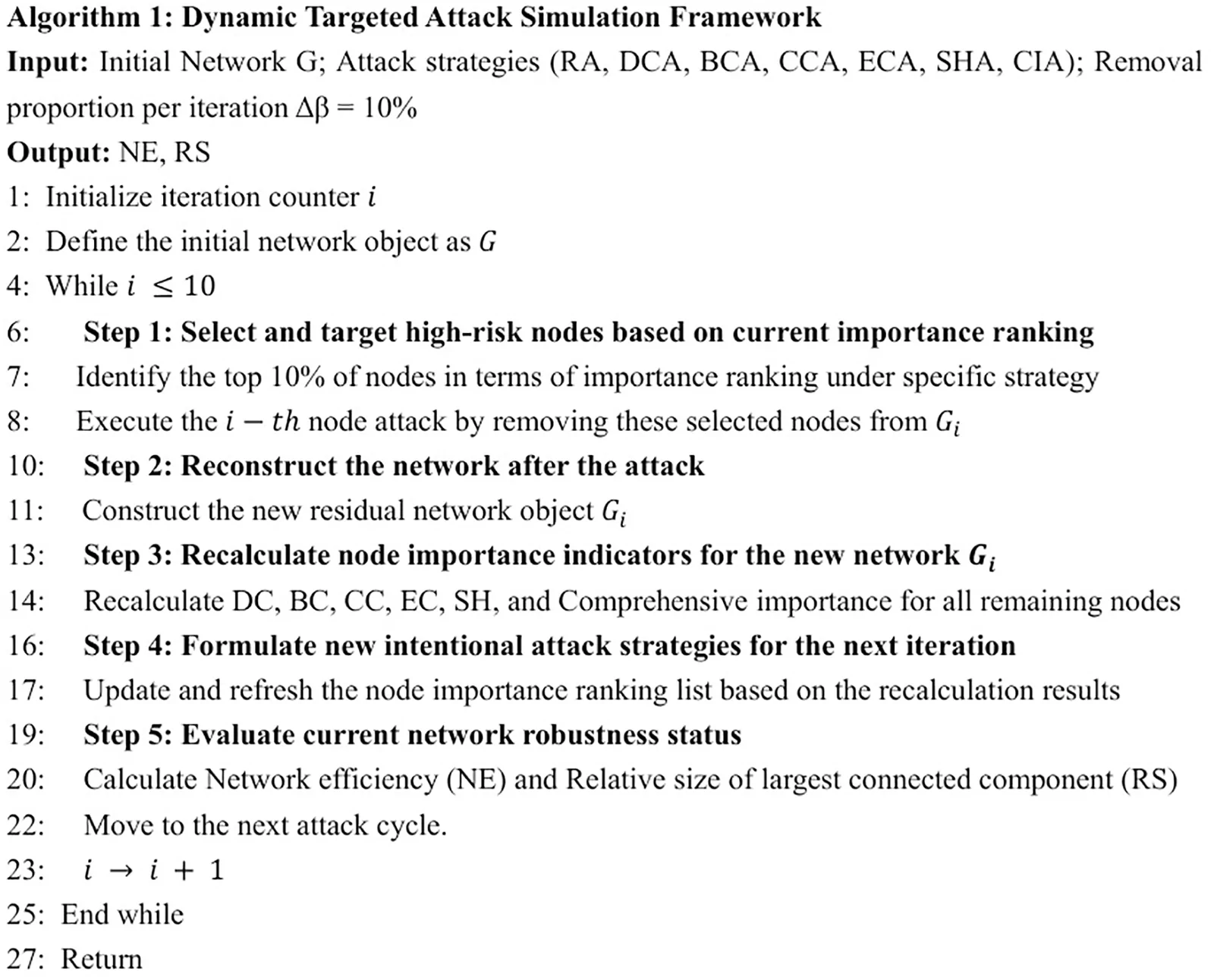
Pseudocode of dynamic network robustness analysis algorithm.

Particularly, this sequential intervention is performed on the network $G_{i}$ after the preceding i−th attack, where each successive step recalculates the importance rankings of the different node indicators within $G_{i}$ to target the top 10% of the remaining nodes. This computational cycle continues until the number of cumulative attacks i triggers a condition where all nodes within the network topology are thoroughly removed, which concludes the dynamic node deletion process. Fig 15 illustrates the comprehensive procedural logic through the pseudocode for the dynamic network robustness analysis algorithm.

**Fig 16:**
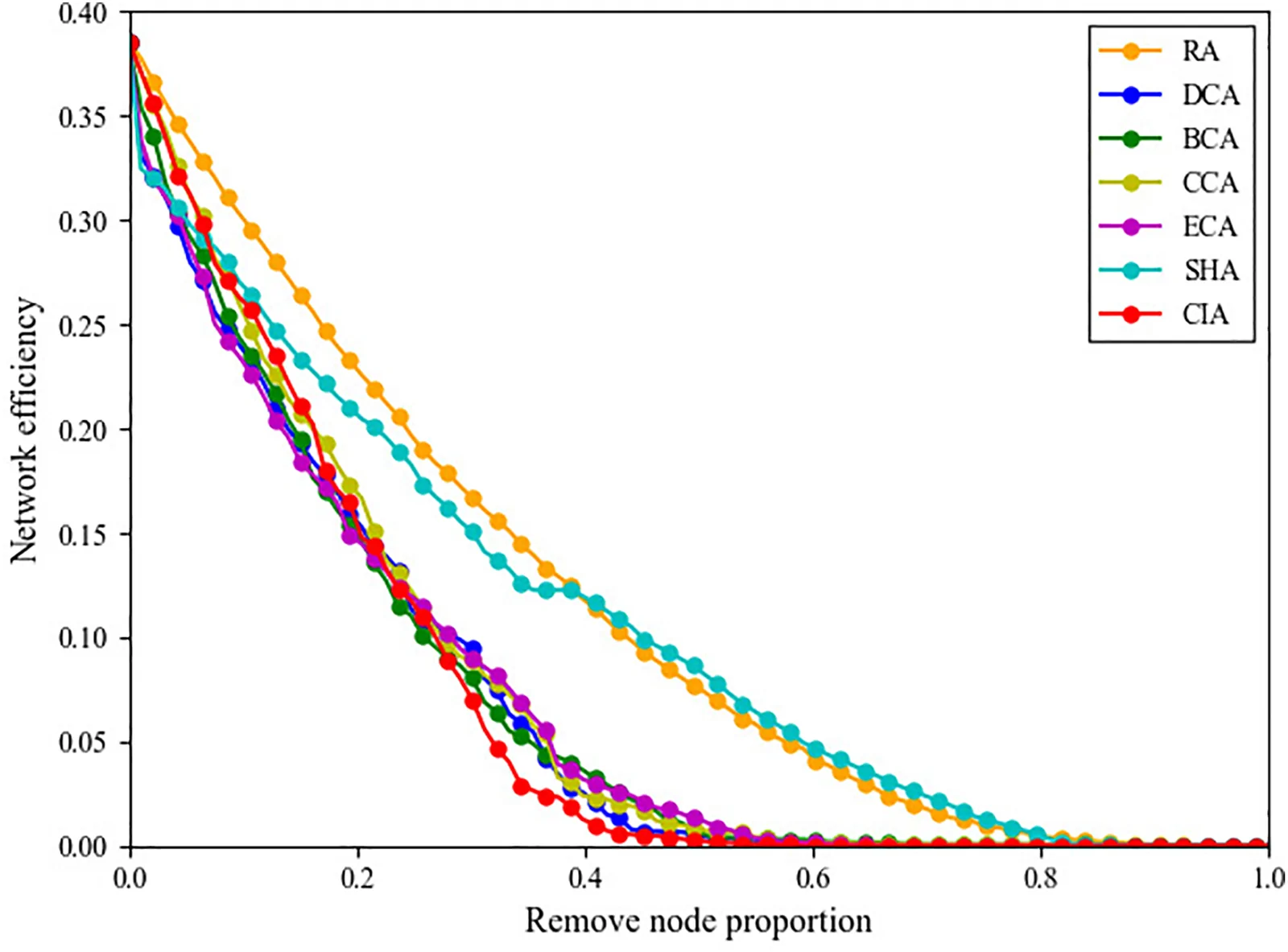
The dynamic change of network efficiency under different attacks.

Fig 16 illustrates the dynamic change of NE under different attacks. From Fig 16, it can be observed that NE gradually decreases with the increase of the node removal ratio β when different attack strategies are employed. Compared to static NE, it is found that under the CIA attack, the NE reaches 0 when the node removal ratio β=0.43. When the node removal ratio β=0.58, the NE under DCA, BCA, CCA, and ECA attacks also drops to 0, indicating network collapse. Throughout the process of node removal, the CIA strategy consistently exhibits the fastest decline in global NE, followed by the DCA strategy. This indicates weaker NE robustness under these two attack strategies. Additionally, the NE curves under different attack strategies show a stepwise decline. Investigation reveals that these nodes play a crucial role in maintaining the ROACN network structure. If these critical nodes are attacked and become ineffective, a large number of isolated points will appear in the network, leading to reduced network connectivity.

**Fig 17:**
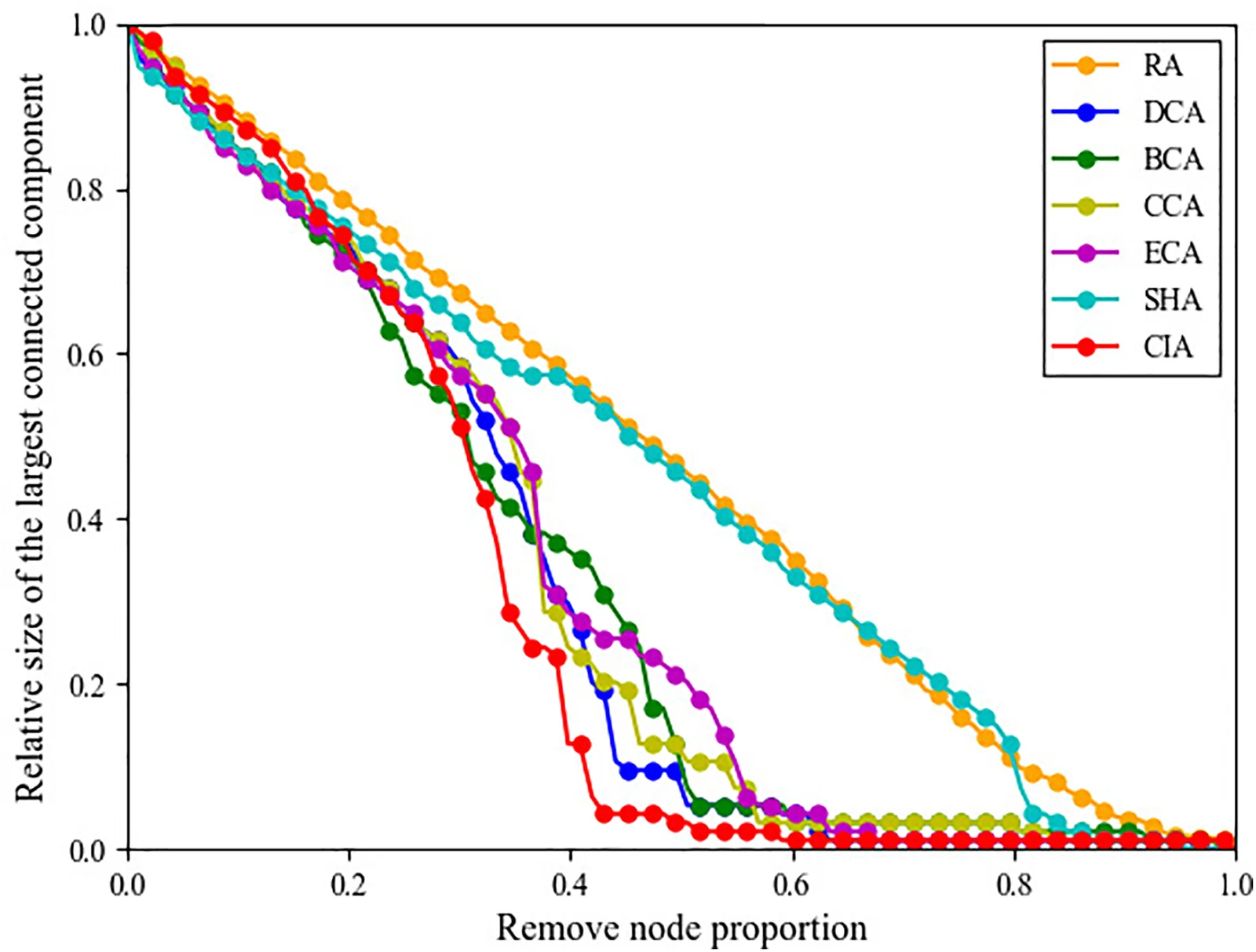
The dynamic change of the relative size of the largest connected component under different attacks.

Fig 17 displays the dynamic change of RS under different attacks. From Fig 17, it can be observed that RS in ROACN decreases as the proportion β of removed nodes increases under different attack strategies. Specifically, comparing different deliberate attack strategies reveals that when β≥0.28, RS under CIA, DCA, BCA, CCA, and ECA attack strategies suddenly decreases rapidly. When β=0.6, the RS value under CIA attack is 0, indicating network collapse at this point. Therefore, when ROACN is subjected to random attack, the network exhibits strong robustness. When ROACN is subjected to deliberate attacks, the network’s connectivity is weaker. Especially under CIA attack, leading to rapid changes in network structure, sharp decline in functionality, and the overall network approaching collapse.

In summary, through the comparative robustness investigations executed across both static and dynamic network configurations, the simulation outcomes demonstrate that the methodology introduced in this study represents the topological importance of nodes within the ROACN with significantly higher fidelity compared to traditional single-metric node indicators. This performance underscores its superior efficacy and pragmatic value specifically within the domain of systemic risk analysis. Additionally, the competitive disruption paradigms exhibit distinct structural characteristics. Although various attack strategies yield divergent degradation trajectories, the optimal destabilization performance consistently arises from rigorous multi-attribute topological analysis rather than arbitrary random failure strategies. When eliminating nodes at an identical removal proportion, targeting and disabling vertices with elevated comprehensive importance values triggers a significantly more rapid and catastrophic reduction in both global network efficiency and the relative size of the largest connected component.

## Discussion

### Suggestions

Based on the final comprehensive importance ranking results, this study selects the top-ranked critical risk factors, including N4, N7, H9, and M8, for targeted discussion and proposes practical and operable risk mitigation strategies. The detailed analyses are presented as follows.

First, brake system failure (N4) ranks first among all identified risk factors, indicating that it acts as a core nodal point triggering multiple railway accident chains. Accordingly, advanced monitoring technologies should be introduced to realize real-time condition monitoring and predictive fault early warning of train brake systems, facilitating the paradigm shift from conventional reactive maintenance to data-driven predictive maintenance. High-precision sensors can be deployed on train braking equipment and integrated into the on-board train control system to achieve proactive fault prediction, which effectively prevents potential safety accidents caused by brake failures.

Second, wheel damage (N7) is verified as a dominant inducement for train derailment accidents. As a core component of the train running gear, the operational condition of wheels directly determines the safety and stability of railway operation. Typical wheel defects mainly include wheel out-of-roundness, tread spalling, and bearing thermal abnormality. It is imperative to adopt technical means to implement real-time dynamic monitoring of wheel status. Meanwhile, priority should be given to wheel inspection and maintenance during scheduled overhaul cycles to curb the generation and propagation of wheel fatigue cracks.

Third, poor communication (H9) serves as a significant human factor contributing to railway accident risks. Smooth and standardized information interaction among multi-post staff is essential for timely identification and disposal of potential operational hazards. In terms of institutional management, oral communication and non-standard professional terminologies in operational scenarios should be strictly prohibited to standardize on-site information transmission. From a technical perspective, natural language processing technology can be applied to assist the generation and issuance of scheduling commands, so as to eliminate human-induced information input errors and improve communication accuracy and efficiency.

Finally, Safety management system not operating (M8) is another critical risk factor that requires high attention. The railway safety management system undertakes core functions including real-time perception of train operating status and external environmental parameters, covering train speed, signal status, rainfall intensity, wind speed, and other key indicators to guarantee operational safety. Therefore, regular maintenance, version iteration, and functional optimization of the safety management system are essential. Furthermore, it is necessary to strengthen the system resilience to enhance its anti-interference capability and fault tolerance under complex and uncertain operating conditions.

### Limitations

The novel risk assessment framework proposed in this study is of great significance to railway operational safety. Nevertheless, several limitations remain in this research.

First, the analysis of topological characteristics of the risk network mainly focuses on nodes, with an emphasis on exploring the importance of different risk nodes. In actual railway operation, risks are interconnected and can propagate along such connections, which makes the topological properties of network edges equally critical. This study fails to conduct systematic quantitative analysis on the correlation mechanism, dynamic evolution characteristics and coupling synergistic effects of risk edges. Accordingly, it cannot fully reveal the accident evolution mechanism induced by the interaction of multiple risks, and its applicability to scenarios with complex risk coupling needs to be further improved.

Second, subjective biases exist in accident text mining and accident chain extraction. The coding of risk factors derived from accident reports relies on manual judgment by domain experts. In addition, human judgment errors are inevitable during the construction of accident chains, resulting in subjective tendencies in sample data and accident chains, and hindering standardized information extraction. Furthermore, the limited number of experts participating in accident text processing is another shortcoming of this study.

## Conclusion

This study utilizes $168$ railway accident reports as primary empirical data sources to identify risk factors and extract causal chains based on foundational accident chain theory. By integrating the topological structural characteristics of complex networks, a railway operation accident complex network model, which is named as the ROACN, is successfully developed. This research focuses on the systematic risk identification and evaluation of the ROACN, wherein a novel comprehensive node importance calculation methodology is designed and the dynamic robustness of the system is meticulously analyzed. This framework accurately identifies key risk nodes and captures the dynamic variations of network characteristics alongside cascading risks during the sequential node failure process. Consequently, this study provides an effective risk identification method for evaluating railway operation systems and establishes a proactive path toward preventing railway accidents. The principal conclusions of this study are organized as follows:

(1) Different from traditional undirected and unweighted network configurations, this methodology extracts risk factors and causal relationships from historical accident documentation via accident chain theory. A directed and weighted complex network model is constructed for railway operation accidents, which possesses the structural capacity to express the precise correlation intensity between different nodes, thereby aiding the mathematical analysis of the risk evolution pathways of railway accidents. Additionally, through the deep analysis of network structural characteristics, the empirical evidence proves that the ROACN operates as a scale-free network. Therefore, utilizing these advanced evaluation indicators within complex networks enables decision-makers to quickly and reliably isolate the root causes of systemic accidents.
(2) To accurately identify the critical risk factors within the ROACN, this study introduces a comprehensive node importance evaluation framework developed through the combinatorial weighting-TOPSIS methodology. This integrated approach harmonizes the respective strengths of the ANP, CRITIC, and TOPSIS models. Furthermore, it employs game theory to optimize the mathematical combination of subjective and objective weights, thereby effectively eliminating the individual bias and imbalances inherent in purely artificial weighting procedures. This hybrid methodology overcomes the inherent limitations of traditional single evaluation indicators and successfully exposes potential key vulnerability nodes hidden deep within complex network structures.
(3) Based on the intrinsic network structural characteristics of the ROACN, seven competitive disruption strategies are deployed for a comprehensive comparative analysis of network robustness. The simulation results indicate that under the static network configuration, the initial network topology reaches a completely paralyzed state when the node removal ratio reaches the threshold of β=0.6. In contrast, under the dynamic adaptive framework, the topology accelerates into a paralyzed state much earlier when the node removal ratio reaches the lower milestone of β=0.4. This stark divergence demonstrates that the ROACN exhibits significantly weaker resistance and structural vulnerability when subjected to dynamic, adaptive node attacks. Additionally, compared with traditional single evaluation indicators, the proposed comprehensive node importance evaluation methodology identifies key risk with significantly higher accuracy, implying that implementing targeted protective measures for these specific nodes provides the most effective leverage for preventing catastrophic railway accidents.

Future work will introduce nonlinear dynamic models to analyze the edge characteristics and dynamic coupling relationships of the risk network. Meanwhile, we will adopt natural language processing (NLP) and multi-expert consensus verification methods to replace pure manual coding.
